# Formulation Stabilization and Disaggregation of Bevacizumab, Ranibizumab and Aflibercept in Dilute Solutions

**DOI:** 10.1007/s11095-018-2368-7

**Published:** 2018-02-28

**Authors:** Steven A. Giannos, Edward R. Kraft, Zhen-Yang Zhao, Kevin H. Merkley, Jiyang Cai

**Affiliations:** Department of Ophthalmology and Visual Sciences, University of Texas Medical Branch, 301 University Blvd, Galveston, Texas 77555 USA

**Keywords:** aflibercept, bevacizumab, ELISA, ranibizumab, SE-HPLC

## Abstract

**Purpose:**

Studies were conducted to investigate dilute solutions of the monoclonal antibody (mAb) bevacizumab, mAb fragment ranibizumab and fusion protein aflibercept, develop common procedures for formulation of low concentration mAbs and identify a stabilizing formulation for anti-VEGF mAbs for use in *in vitro* permeation studies.

**Methods:**

Excipient substitutions were screened. The most stabilizing formulation was chosen. Standard dilutions of bevacizumab, ranibizumab and aflibercept were prepared in PBS, manufacturer’s formulation, and the new formulation. Analysis was by SE-HPLC and ELISA. Stability, disaggregation and pre-exposure tests were studied.

**Results:**

When Avastin, Lucentis and Eylea are diluted in PBS or manufacturer’s formulation, there is a 40–50% loss of monomer concentration and drug activity. A formulation containing 0.3% NaCl, 7.5% trehalose, 10 mM arginine and 0.04% Tween 80 at a pH of 6.78 stabilized the mAbs and minimized the drug loss. The formulation also disaggregates mAb aggregation while preserving the activity. Degassing the formulation increases recovery.

**Conclusions:**

We developed a novel formulation that significantly stabilizes mAbs under unfavorable conditions such as low concentration or body temperature. The formulation allows for tissue permeation experimentation. The formulation also exhibits a disaggregating effect on mAbs, which can be applied to the manufacture/packaging of mAbs and bioassay reagents.

**Electronic supplementary material:**

The online version of this article (10.1007/s11095-018-2368-7) contains supplementary material, which is available to authorized users.

## Introduction

The commercial development of therapeutic monoclonal antibodies commenced in the early 1980’s, and by 1986 the first therapeutic monoclonal antibody (mAb), Orthoclone OKT3, was approved for the prevention of kidney transplant rejection. (1) As of 2015, the highly dynamic late-stage commercial pipeline of recombinant therapeutics now includes nearly 50 molecules. (2) The majority of approved antibody drugs are used to treat cancer and inflammation. However, two of these monoclonal antibodies, bevacizumab (Avastin^®^ and ranibizumab (Lucentis^®^), and the fusion protein aflibercept (Eylea^®^) show anti-VEGF properties and are used to treat neovascular (wet) age-related macular degeneration (AMD) and diabetic retinopathy. (3) We chose to concentrate our studies on ranibizumab, which is FDA approved for AMD, while also studying bevacizumab (off-label use) and FDA approved aflibercept as additional anti-VEGF agents, which are also used for AMD treatment.

One of the major complications of manufacturing and working with mAbs is the tendency for mAb aggregation. (4–6) Hydrophobic areas on the surface amino acid sequence are thought to be the most likely location seeding aggregation. Aggregation represents the most common form of instability in protein drugs. (7) Aggregation, in which two or more monomeric units of mAb may bind to each other, is considered an undesirable phenomenon that leads to a decrease in available efficacious product, potentiating the immunogenicity and other side effects in patients. (8,9) Moreover, protein aggregation can be induced by many factors during or after manufacturing, such as physical stresses, elevated temperatures or even simple agitation during shipping. (10)

Due to their proteinaceous composition, antibodies present generic formulation issues that are similar for most protein therapeutics. (5) Various strategies have been developed over the years to counteract protein degradation and aggregation. (11) Briefly these fall into the categories of looking at pH, buffer and excipients to try to reduce aggregation by protein folding or by changing the surface attraction potential. In the area of excipients, non-reducing sugars, amino acids and surfactants are used to stabilize the mAb. (12–14)

Usually, mAbs are formulated in high concentrations (generally 1 mg/ml – 60 mg/ml). (15) Subcutaneous injection preparations are ultra-high-concentration formulations ranging from 75 to 200 mg/ml. (16) Little is known about protein solution dynamics in dilute solutions below 1 mg/ml. Only recently has attention turned to the investigation of antibody stability of low-dose antibodies as well as clinical dilutions of mAb medications. (17) We became interested in the stability of dilute mAb solutions when we were developing and validating methods for mAb *in vitro* transscleral permeation using traditional two-compartment Franz permeation cells.

The study of dilute mAb solutions is also important to clinical applications. High concentration mAb solutions are regularly diluted in saline for IV dosing. (17,18) Not only is the diluent composition a factor for aggregation, but we have also found that the dilution itself is also very important. We have found that bevacizumab, ranibizumab and aflibercept are very prone to diminished function once removed from their manufacturer’s vial and diluted. Prior research by others determining mAb drug concentration in human fluid samples, as well as *in vitro* permeation studies, have utilized ELISA assay kits for quantification. (19) High sensitivity ELISA methods require drug samples to be diluted to bring the samples within the usable dynamic range of the method, generally 1-1000 ng/ml, more typically 1-100 ng/ml. We have extended the dynamic range to 4–144,000 ng/ml with SE-HPLC and correlated and validated the two analytical methods. Frequently PBS is used as the sample diluent. We have found that mAb dilution with PBS causes a decrease of monomer concentration that may have led to a less than actual representation of drug concentrations in ELISA assayed solution in various *in vitro* and *in vivo* studies. This phenomenon has recently been reported by others as well. An additional factor we uncovered was the issue of degassing. Finally, aggregated mAbs were found to be disaggregated when introduced into our formulation.

## Materials and Methods

### Materials

L-Argininine, L-(+)-Glutamic Acid, L-Histidine HCl, sodium phosphate dibasic anhydrous, sodium phosphate monobasic monohydrate, sodium sulfate anhydrous, polysorbate 20 (Tween^®^ 20), phosphate buffered saline (10X), and HPLC water were obtained from Fisher Scientific, Fair Lawn, NJ. Pluronic^®^ F 127, α,α-trehalose dihydrate, polysorbate 80 (Tween^®^ 80, low peroxide), and sodium chloride, USP was purchased from Sigma-Aldrich, St Louis Mo and normal saline 0.9% obtained from Baxter Healthcare Corp., Deerfield, IL.

ELISA analytical kits for bevacizumab (kit #AVA-E-U51) and ranibizumab (kit #LUC-E-U52) were obtained from United Immunoassay Inc., San Bruno, CA USA. Aflibercept was analyzed using an ELISA procedure as described Celik *et al.* (20)

Avastin^®^ (bevacizumab) 25 mg/ml and Lucentis^®^ (ranibizumab) 10 mg/ml were obtained from Genentech, South San Francisco CA. IAI Eylea^®^ (intravitreal aflibercept injection) 48.2 mg/ml was obtained from Regeneron, Tarrytown NY. (Note: bulk aflibercept as provided by Regeneron is at 48.2 mg/ml while the common pharmaceutical preparation is formulated at 40.0 mg/ml). All other reagents were of analytical or USP purity.

### Sample Analysis

#### Enzyme-Linked Immunosorbent Assay (ELISA)

Bevacizumab and ranibizumab ELISA assays were performed as per manufacturer’s instructions except for a substitution of the base analytical standard diluent material which was taken from the pharmaceutical preparations obtained and diluted as described below. Aflibercept ELISA was performed as described by Celik *et al.* (20), except for a substitution of the base analytical standard diluent material which was taken from the pharmaceutical preparations obtained and diluted as described below. Drug standard dilutions were diluted with the manufacturer’s diluent solution (or as described by Celik for aflibercept), PBS, as well as with the new formula, as described below. Standard concentration curves were generated for each of the diluent groups. The ELISA method dynamic ranges are as follows: bevacizumab 1–281.25 ng/ml, ranibizumab 1-100 ng/ml and aflibercept 1-100 ng/ml.

#### Size-Exclusion High Performance Liquid Chromatography (SE-HPLC)

Analytical size-exclusion chromatography was performed using an Agilent HPLC system HP1100 from Agilent Technologies (Santa Clara, CA) with a UV detector. A TSKgel SuperSW mAb HTP 4.6 mm × 15 cm, 4 μm SEC column with TSKgel guard column (Tosoh Bioscience LLC, King of Prussia, PA) was used in the early studies of bevacizumab and the ranibizumab formulation studies. The HTP column was equilibrated at a flow rate of 0.4 ml/min using HPLC mobile phase (100 mM sodium sulfate in 100 mM phosphate buffer (pH 6.53) in HPLC water.

We started with a newly developed mAb high-throughput (HTP) SE-HPLC from Tosoh Bioscience. The general strategy for tissue permeation studies is to use a shorter column in order to reduce reagent volumes and reduce analytical cycle time for the numerous samples generated. This column worked well for the early experiments with bevacizumab and the formulation studies using ranibizumab. Aflibercept was received much later. Aflibercept was difficult to separate and resolve from the excipient peak using a short column. We found that using a longer column (same manufacturer, class and packing material) separated the aflibercept from the excipient component and allowed us to measure the aflibercept concentrations.

The remaining studies, including aflibercept, were performed using a TSKgel UltraSW Aggregate 7.8 mm × 30 cm, 3 μm column with TSKgel UltraSW guard column (Tosoh Bioscience LLC, King of Prussia, PA). Mobile phase, comprising 85% 100 mM sodium sulfate in 100 mM phosphate buffer in HPLC water (adjusted to pH 6.68) with 15% acetonitrile/0.1% trifluoroacetic acid, was used at a flow rate of 0.6 ml/min. Sample injections were 100 μl in volume. The eluted protein was monitored by UV Absorbance at 212 nm. The lower limit of detection (LOD) was 2.19 ng/ml and the lower limit of quantification (LOQ) was 8.79 ng/ml for both bevacizumab and ranibizumab. The LOD and LOQ for aflibercept were 8.79 ng/ml and 17.578 ng/ml respectively. Silanized HPLC sample vials and silanized vial inserts from Agilent Technologies (Santa Clara, CA), were used throughout.

### *In Vitro* Experiments

#### Effect of Carrier Matrix Composition

When phosphate buffered saline (PBS) was used as a diluent, we found that the monomeric bevacizumab significantly decreased. In a subsequent assay, serial dilutions of bevacizumab were prepared in PBS and assayed by SE-HPLC at 0 time *vs* the same samples analyzed 3 h when kept at room temperature (23°C), in order to check the amount of monomer decrease due to PBS. Serial dilutions (1:1, e.g. 500 μl:500 μl) were performed using low binding pipette tips with Eppendorf Protein LoBind^®^ tubes. This method provided a large volume to surface ratio and minimal pipetting errors. We then placed the samples in silanized glass vial inserts in HPLC vials to carry out the SE-HPLC experiments. After the first SE-HPLC analysis of samples from the silanized glass HPLC inserts, the same samples remained in the same silanized glass inserts for 3 h at room temperature (in the HPLC sample holder). There was no secondary container, aliquot or transfer involved. These same samples, from the same vials, were then re-analyzed using the same SE-HPLC under the same conditions.

#### Formulations

The experimental design for the formulations to be tested for improved stability started with the manufacturer’s formulations for ranibizumab and bevacizumab. Bevacizumab (Avastin^®^) and ranibizumab (Lucentis^®^) have different formulations from the manufacturer. Bevacizumab (Avastin^®^) is formulated from the manufacturer at a concentration of 25 mg/ml in a solution of 6% (60 mg/ml) α, α-trehalose dihydrate, 0.04% (0.4 mg/ml) polysorbate 20 (Tween^®^ 20), 0.58% (5.8 mg/ml) sodium phosphate (monobasic, monohydrate), 0.12% (1.2 mg/ml) sodium phosphate (dibasic, anhydrous), in water at pH 6.2. (21)

Ranibizumab (Lucentis^®^) is formulated from the manufacturer at a concentration of 10 mg/ml in a solution of 10% (100 mg/ml) α, α-trehalose dihydrate, 0.01% (0.1 mg/ml) polysorbate 20 (Tween^®^ 20), and 10 mM (1.98 mg/ml) L-histidine in water for injection at a pH 5.5. (22)

Based upon the manufacturer’s formulation, excipient substitutions were screened with dilutions of standard concentrations of ranibizumab, as explained in the Supplemental Section. These are shown in Table I. Water, PBS, saline (0.9% NaCl) and 0.3% NaCl were used as the starting solutions. Other than the manufacturer’s formulation, sodium phosphate buffer was used in all of the formulations. Trehalose was used throughout the formulations, owing to its protein stabilizing quality. The amino acids arginine, histidine and glutamic acid were tested in various capacities. Surfactants were also tested. The manufacturer’s formulation contained 0.01% Tween 20. Concentrations of 0.01% and 0.04% of Tween 20 were screened. Another surfactant, Tween 80, was tested at a concentration of 0.04%. Additionally, Pluronic127 was tested. Finally, pH was looked at as a variable in the formulations. These were then analyzed by SE-HPLC. Formula 14 (F14), comprising 7.5% α,α trehalose dihydrate, 100 mM sodium phosphate, 10 mM L-arginine, 0.3% sodium chloride, 0.04% polysorbate 80 with the balance being ultrapure water (Milli-Q, EMD Millipore USA) at a pH of 6.78 was chosen for further analysis.

**Table I:** Ranibizumab Formulations Tested

ID.	H_2_O	0.9% NaCl^a^	0.3% NaCl	Trehal. %	Arg mM	Glu mM	His mM	T-20%	T-80%	P-127%	pH
1	X			10			10	0.01			5.50
2	X	X		10			10	0.04			7.40
3	X	X		10			10	0.01			7.40
4	X	X		5			5	0.04			7.40
5	X	X		5				0.04			7.40
6	X	X		10				0.04			7.40
7	X	X		5			10	0.04			6.53
8	X	X		10				0.04			6.53
9	X	X		10	10			0.04			6.81
10	X		X	10	10			0.04			6.81
11	X		X	10	10	10				1	6.81
12	X		X	5	75	75		0.04			6.61
13	X		X	10	10				0.04		6.78
14	X		X	7.5	10				0.04		6.78

In the above Table I, Trehal. = Trehalose, T-20 = Tween 20, T-80 = Tween 80 and P-127 = Pluronic-127.

Formulations 10–14 made with 100 mM phosphate buffer.

^a^Phosphate buffered saline

Ranibizumab dilutions were made in F14 at pH values from 6.78 to 7.4. F14 preparations at pH values of 6.78, 7.0, and 7.4 were prepared and used as a comparison to determine the effects of pH on SE-HPLC area. Ranibizumab 10 mg/ml was first diluted to 144,000 ng/ml and then further serially diluted in 1:1 concentration dilution steps from 72,000 ng/ml to 8.789 ng/ml. Diluted samples from 8.789 ng/ml to 18,000 ng/ml were then analyzed by Size Exclusion HPLC (SE-HPLC) as previously described. A usable analytical range of 140.625 to 18,000 ng/ml was selected.

#### ELISA/SE-HPLC Validation Study

Antibody standard serial dilutions from the pharmaceutical preparations were made in a range from 144,000 ng/ml to 1.0985 ng/ml in 1:1 steps using F14. Additional dilutions for bevacizumab at 40.96, 64, 80, 100 and 200 ng/ml were made from the serial dilution steps. Additional dilutions of 100 ng/ml of ranibizumab and aflibercept at 100 ng/ml were made from the serial dilutions steps. Additional dilutions were made to accommodate the narrow dynamic range of ELISA kits and to generate more standard curve data points for the respective ELISA methods. Dilution standards were divided into two parts for ELISA and SE-HPLC analysis. The same procedure was followed to prepare and analyze the antibody standard serial dilutions in PBS.

#### Stability Study

Specific formulations of ranibizumab were tested for stability. Sets of standard dilutions ranging from 3.9 ng/ml to 6000 ng/ml were prepared from Formulas 1 and 14. Formula 1 is the manufacturer’s formula and Formula 14 is the new formulation. These solutions were divided in half and one set was analyzed as time 0 and the other set was incubated at 37°C for 24 h. Both sets were analyzed by SE-HPLC.

#### Disaggregation and Pre-Exposure Studies

Bevacizumab**,** ranibizumab and aflibercept were used as model antibodies to determine the anti-aggregation or “disaggregation” potential of F14. Ranibizumab and aflibercept were also used as model antibodies to determine the reduced potential of aggregation by “pre-exposure” to F14 prior to dilution with a carrier matrix with little anti-aggregation potential.

For the “disaggregation” evaluation, a disaggregation test was run in order to investigate the reversibility of mAb aggregation with F14. For this test, bevacizumab and ranibizumab were diluted into PBS (100μg/ml) and stored overnight (17 h), to initiate aggregation. These solutions were then diluted with F14 and with PBS as controls.

For the “disaggregation” evaluation, aflibercept 48.2 mg/ml was diluted in F14 to a concentration of 144 μg/ml as a positive control. Aflibercept 48.2 mg/ml was diluted in PBS to a concentration of 144 μg/ml as a negative control. Disaggregation potential was assessed by subsequent dilution of the 144 μg/ml PBS by F14 from 72,000 ng/ml to 2.197 ng/ml. The positive control F14 and the negative control PBS were also diluted with the respective diluents 72,000 ng/ml to 2.197 ng/ml. Samples were analyzed by SE-HPLC; peak area values were plotted against the concentration and slope values were determined.

For “pre-exposure” to prevent aggregation evaluation, ranibizumab 10 mg/ml was diluted in F14 to a concentration of 144 μg/ml as a positive control. Ranibizumab 10 mg/ml was diluted in PBS to a concentration of 144 μg/ml as a negative control. Pre-exposure anti-aggregation potential was assessed by subsequent dilution of the 144 μg/ml F14 with PBS from 72,000 ng/ml to 2.197 ng/ml. The positive control F14 and the negative control PBS were also diluted with the respective diluents 72,000 ng/ml to 2.197 ng/ml. Samples were analyzed by SE-HPLC; peak area values were plotted against the concentration and slope values were determined.

For “pre-exposure” to prevent aggregation evaluation, aflibercept 48.2 mg/ml was diluted in F14 to a concentration of 144 μg/ml as a positive control. Aflibercept 48.2 mg/ml was diluted in PBS to a concentration of 144 μg/ml as a negative control. Pre-exposure anti-aggregation potential was assessed by subsequent dilution of the 144 μg/ml F14 with PBS from 72,000 ng/ml to 2.197 ng/ml. The positive control F14 and the negative control PBS were also diluted with the respective diluents 72,000 ng/ml to 2.197 ng/ml. Samples were analyzed by SE-HPLC; peak area values were plotted against the concentration and slope values were determined.

#### Statistical Analysis

Data is presented as mean ± Standard Deviation (SD). Group to group analysis was conducted with student’s two-tailed t-test using Microsoft Excel. In all cases, a *p* value <0.05 was considered to be significant. Pearson product-moment correlation coefficient calculations were performed using SigmaPlot (Systat Software Inc., San Jose, California).

## Results

### *In Vitro* Experiments

#### Conventional Carrier Matrix Failed to Preserve Monomeric mAb *Versus* Time and Temperature

**Fig. 1:**
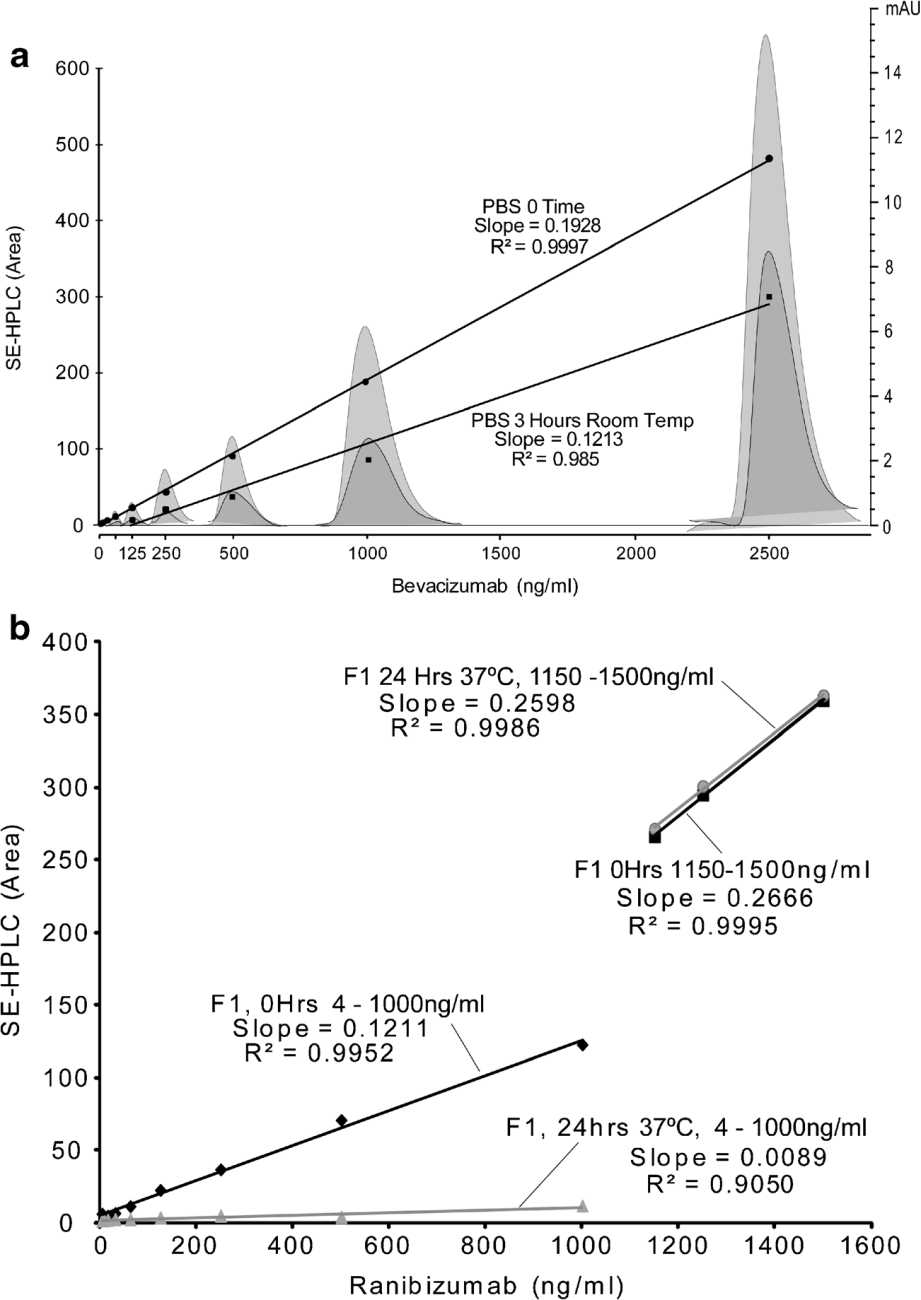
Monomeric bevacizumab significantly decreases when phosphate buffered saline (PBS) is used as a diluent. Representative SE-HPLC chromatograms showing bevacizumab in PBS at time 0 and after 3 h and graphical representation of SE-HPLC area and the slope for bevacizumab diluted with PBS at time 0 *versus* 3 h at room temperature (**a**), graphical representation of SE-HPLC area and the slope of the effect of dilution on monomer concentration of ranibizumab over time at 37°C in the manufacturer’s formulation; formula 1 (**b**). Shown are time 0 and after 24 h storage at 37°C.

We initially found that monomeric bevacizumab significantly decreased when phosphate buffered saline (PBS) was used as a diluent. We then ran an assay, in which serial dilutions of bevacizumab were prepared and assayed by SE-HPLC at time 0 *vs* the same samples analyzed 3 h later, when kept at room temperature (RT, 23°C), in order to check the amount of monomer decrease due to PBS. The monomeric bevacizumab was found to decrease within the 3 h (Fig. 1a), with the starting standards having a SE-HPLC area slope of 0.1928 and then 3 h later having a SE-HPLC area slope of 0.1213; a loss of about 40%. The estimated fraction % of the manufacturer’s composition after dilution with PBS are presented in the Supplemental Section.

We next tested if diluting with the manufacturer’s formula (F1) would prevent ranibizumab from losing monomer concentration. Same as that observed in bevacizumab, we noted a significant decrease of monomer concentration at 24 Hrs. under both RT and body temperature (BT) when diluted in the F1 within the concentration range of 1000 ng/ml (Fig. 1b). Interestingly, there was a concentration discontinuity between 1000 ng/ml and 1150 ng/ml (Fig. 1b); better recovery rate was observed in the high end range compared with the lower range, indicating that a higher stock concentration improved the stability of mAb monomers.

#### The Optimal Formulation for Preserving Functional mAb Monomer

**Fig. 2:**
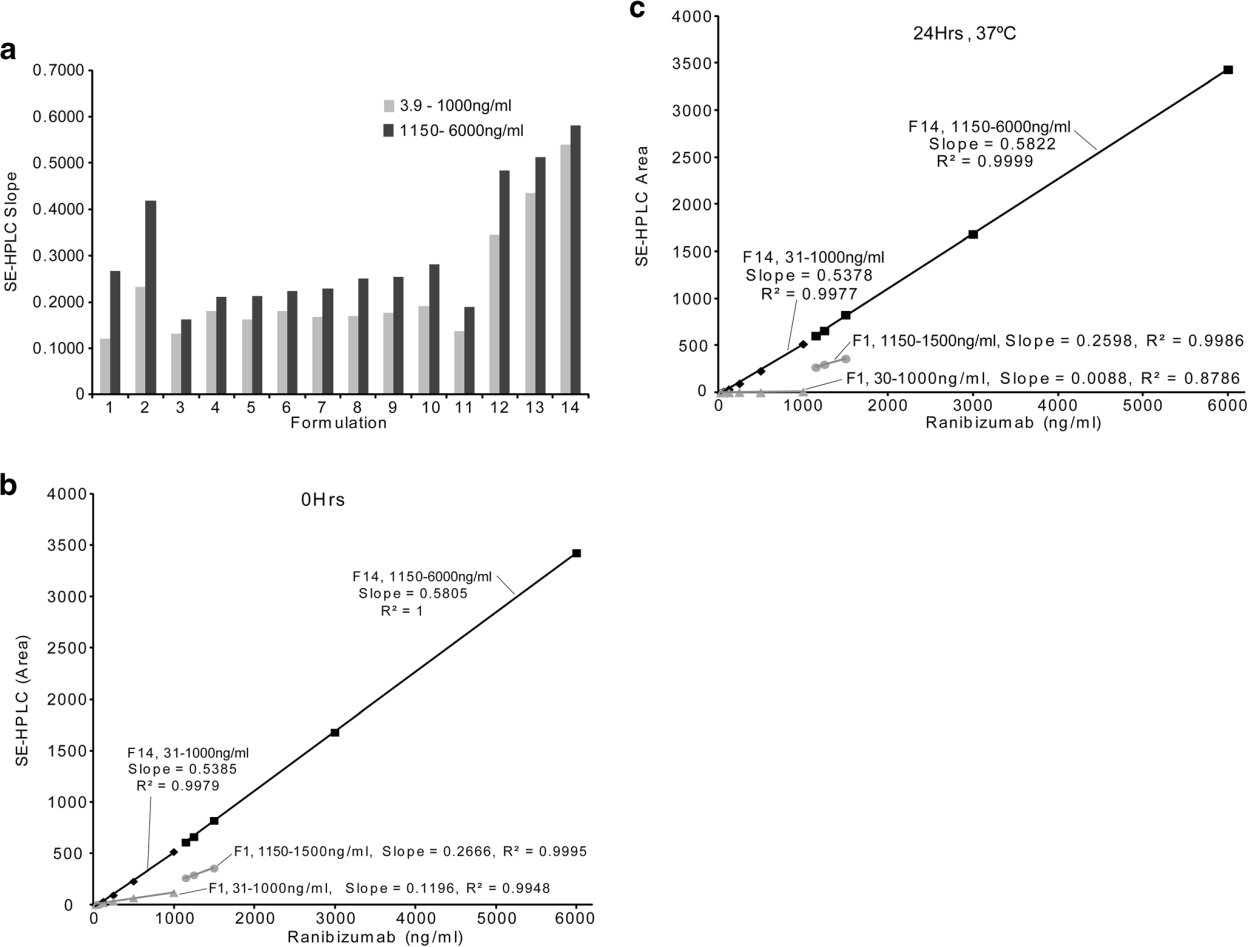
Graphical representation of the formulations of Table I (**a**). The slope of the effect of dilution on monomer concentration of ranibizumab, as measured by SE-HPLC, is shown in b and c. Ranibizumab, diluted in the same formulation used by the manufacture, formula 1, shows an immediate 40% loss of monomer concentration, as compared to Formula 14 (**b**, **c**). Fig. 2c shows that heating to body temperature significantly produces more degradation.

Noticing that storage formulations affected the mAb aggregation, we next experimented on different formulations in order to find the one that best preserved the monomer concentration. Among the tested combinations (Table I), Formulations 13 and 14 showed the best preservation of the monomer concentration by higher and nearly continuous concentration slope values as analyzed by SE-HPLC (Fig. 2a and Table II). Additionally, Formula 14 (F14) had the least difference between the lower concentration slope of 0.5385 and the higher concentration slope of 0.5805 giving an almost linear standard curve for concentrations 3.9 ng/ml – 6000 ng/ml.

**Table II:** Slopes and Respective r^2^ Values of the Formulas of Fig. 2a

Formula	HPLC Slope (3.9-1000 ng/ml)	R^2^	HPLC Slope (1150-6000 ng/ml)	R^2^
1	0.1211	0.9952	0.2666^a^	0.9995
2	0.2321	0.9971	0.4189^a^	0.9940
3	0.1317	0.9990	0.1618	0.9987
4	0.1808	0.9977	0.2104	0.9996
5	0.1628	0.9994	0.2126	0.9994
6	0.1808	0.9989	0.2244	0.9999
7	0.1681	0.9994	0.2297	0.9982
8	0.1697	0.9966	0.2510	0.9991
9	0.1766	0.9976	0.2549	0.9995
10	0.1908	0.9960	0.2819	0.9987
11	0.1363	0.9941	0.1888	1.0000
12	0.3440	0.9983	0.4843	0.9992
13	0.4343	0.9989	0.5124	1.0000
14	0.5385	0.9979	0.5805	1.0000

^a^1150 ng/ml - 1500 ng/ml

To further exploit the effect of F14 under biological conditions, we ran a series of stability tests based on ranibizumab at 37°C. When diluted in F1, ranibizumab showed an initial slope of 0.1196 under the concentration below 1150 ng/ml while demonstrating a slope of 0.2666 when above 1150 ng/ml. After exposure to 37°C for 24 h, the ranibizumab all but disappears in the lower concentrations with a slope of 0.0088. The concentrations at 1150 ng/ml and above are detected and with a slope of 0.2598 (Table III). When using F14, the critical concentration point at 1000 to 1150 ng/ml is much less evident before and after exposure to 37°C. A significant higher slope value for F14 was noted comparing to F1 (Fig. 2b and c).

**Table III:** Results of Ranibizumab Stability Study

Formula	HPLC Slope (31-1000 ng/ml)	R^2^	HPLC Slope (1150-6000 ng/ml)	R^2^
1				
0 Hrs.	0.1196	0.9948	0.2666^a^	0.9995
24Hrs.	0.0088	0.8786	0.2598^a^	0.9986
14				
0 Hrs.	0.5385	0.9979	0.5805	1.0000
24 Hrs.	0.5378	0.9977	0.5822	0.9999

^a^1150ng/ml - 1500 ng/ml

Several of the formulations were tested for stability. Sets of ranibizumab standard dilutions ranging from 3.9 ng/ml to 6000 ng/ml were prepared from formulations 1 and 14. Stability tests were conducted at 37°C for 24 h. These were then analyzed by SE-HPLC (see Table III).

#### F14 Is Protective to Other Anti-VEGF Agents from Losing Monomers

**Fig. 3:**
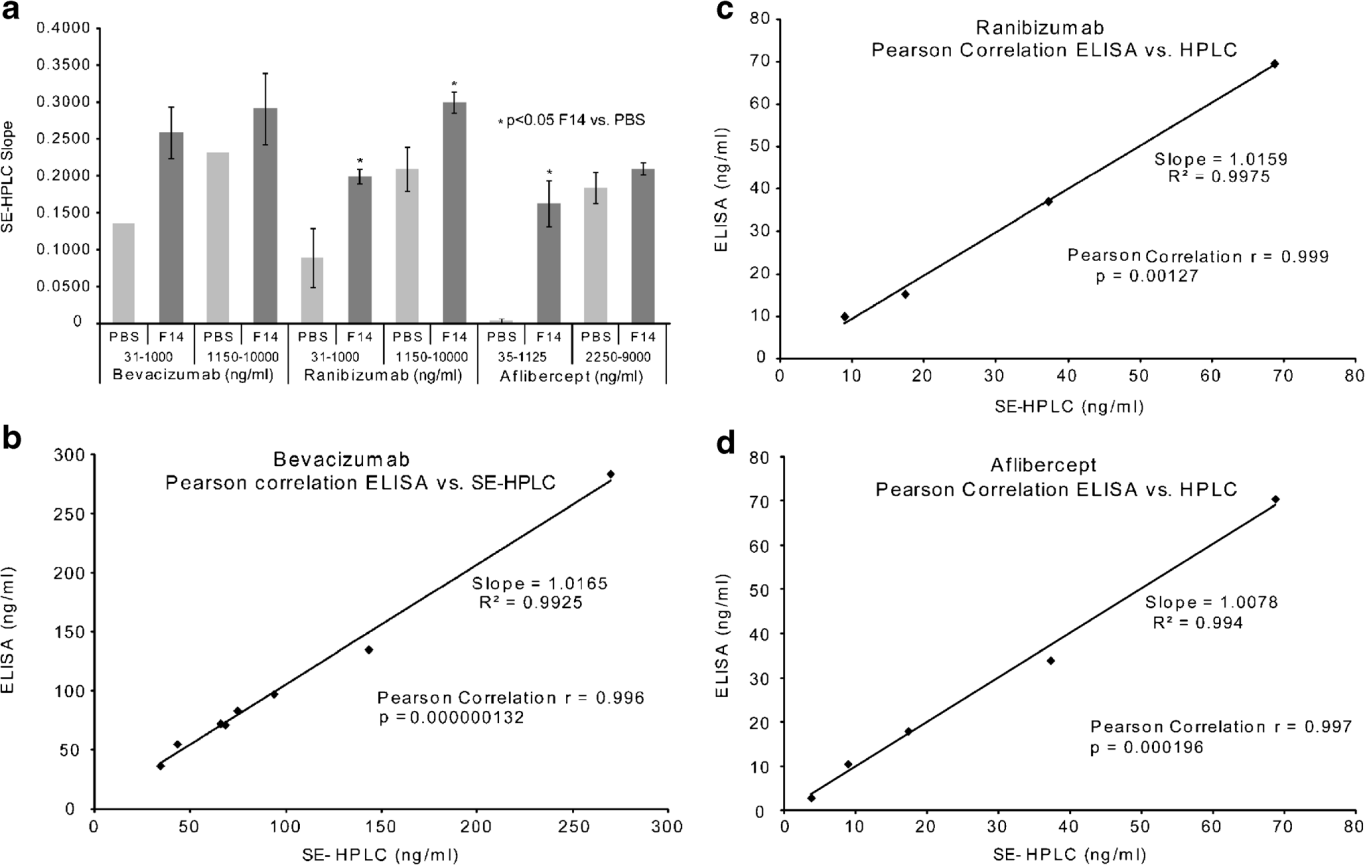
Results of studies of bevacizumab, ranibizumab and aflibercept, diluted with PBS, and compared to dilutions with formula 14 at room temperature (23°C) (**a**). Correlation between enzyme-linked immunoassay (ELISA) and size exclusion high performance liquid chromatography (SE-HPLC) analytical methods (**b**, **c**, **d**). Figs. 6b, 6c and 6d provide graphical representation of bevacizumab, ranibizumab and aflibercept respectively. The data plots show the Pearson Moment Correlation coefficient r value.

Expectedly, F14 was protective to other mAbs aside from ranibizumab. Across these mAbs, bevacizumab has a high monomer recovery in F14 with a SE-HPLC slope of 0.2590 ± 0.0353 for the lower concentration and 0.2915 ± 0.0485 for the higher concentration (Fig. 3a). Meanwhile, using PBS as diluent was detrimental to bevacizumab monomers for both the low and higher concentrations, respectively. Similarly, ranibizumab had a high monomer recovery in F14 with 0.1995 ± 0.0099 (*p* < 0.005) for the lower concentration and 0.2996 ± 0.0145 (*p* < 0.005) for the higher concentration comparing with PBS group. The best protection was seen with aflibercept monomer, which practically disappeared when diluted in PBS at the lower concentrations, but retained a SE-HPLC slope of 0.1529 ± 0.0297 with F14 (*p* < 0.01). At the higher concentration range, F14 still protects aflibercept monomer with a slope of 0.2100 ± 0.0081, whereas with PBS there is less monomer recovery with slope of 0.1839 ± 0.0213. In addition, the protective action of F14 was viable under different pH conditions as no difference in monomer concentration was noted at 3 different pH values (Table IV). This provides the flexibility for manufacturers to fit the optimal pH requirements for individual molecules.

**Table IV:** Results of the Ranibizumab/Formula 14 pH Study

Antibody	pH 6.78		pH 7.0		pH 7.4	
	HPLC Slope	R^2^	HPLC Slope	R^2^	HPLC Slope	R^2^
Ranibizumab						
140-1000 ng/ml	0.1719	0.9963	0.1880	0.9913	0.1813	0.9806
1125-18,000 ng/ml	0.2166	0.9958	0.2267	0.9953	0.2171	0.9959

To further confirm our finding, we compared our HPLC assay with ELISA. For bevacizumab, Pearson product-moment correlation coefficient analysis was run on the paired values demonstrating that the ELISA and SE-HPLC F14 derived values are closely and significantly correlated, with a Pearson correlation of r = 0.996, *p* < 0.001, (Fig. 3b).

For ranibizumab, (Fig. 3c), standard dilutions of 8.789, 17.578, 35.156 and 70.31 ng/ml were analyzed by ELISA and SE-HPLC. Pearson correlation analysis was run on the paired values demonstrating that the ELISA and SE-HPLC derived values are closely and significantly correlated, with a Pearson Correlation of r = 0.999.

For aflibercept, (Fig. 3d), standard dilutions of 4.3945, 8.789, 17.578, 35.156 and 70.31 ng/ml were analyzed by ELISA and SE-HPLC. Pearson correlation analysis was run on the paired values demonstrating that the ELISA and SE-HPLC derived values are closely and significantly correlated, with a Pearson Correlation of r = 0.997.

#### Disaggregation and Pre-Exposure Studies

A disaggregation study was run in order to test the reversibility of mAb aggregation with Formula 14. Bevacizumab, ranibizumab and aflibercept were placed in PBS overnight to allow for aggregation. They were then diluted in F14 in order to test the ability of F14 to return the aggregated mAbs to monomer status. The results from the disaggregation study are shown in Table V, taking into account the slope discontinuities at 1000 ng/ml.

**Table V:** Results of Disaggregation Study Showing SE-HPLC Slopes of Bevacizumab, Ranibizumab and Aflibercept

Anti-VEGF Agent	PBS Control HPLC Slope	Formula 14 Control HPLC Slope	Recovery of aggregated PBS/antibody with F14 HPLC Slope
Bevacizumab			
31-1000 ng/ml	0.1361	0.2961	0.2645
1150-10,000 ng/ml	0.2314	0.3510	0.3170
Ranibizumab			
31-1000 ng/ml	0.0541	0.1751	0.1549
1150-10,000 ng/ml	0.1729	0.2892	0.2696
Aflibercept			
17-1000 ng/ml	0.0151	0.2891	0.3299
1125-9000 ng/ml	0.3541	0.3454	0.3906

As seen in Table V, bevacizumab in PBS has a slope of 0.1361 for the lower concentrations and a slope of 0.2314 for the higher concentrations. However, when the bevacizumab/PBS is introduced to F14, the recovery rebounds with a slope of 0.2645 for the lower concentrations and 0.3170 for the higher concentrations. These values are close to the slopes of 0.2961 and 0.3510, which are the slopes representing bevacizumab, when placed in F14 from the beginning.

Ranibizumab in PBS has a slope of 0.0541 for the lower concentrations and a slope of 0.1729 for the higher concentrations. However, when the ranibizumab/PBS is introduced to F14, the recovery rebounds with a slope of 0.1549 for the lower concentrations and 0.2696 for the higher concentrations. These values are close to the slopes of 0.1751 and 0.2892, which are the slopes representing ranibizumab, when placed in F14 from the beginning.

Aflibercept in PBS has a slope of 0.0151 for the lower concentrations and a slope of 0.03541 for the higher concentrations. However, when the aflibercept/PBS is introduced to F14, the recovery rebounds with a slope of 0.3299 for the lower concentrations and 0.3906 for the higher concentrations. These values are close to the slopes of 0.2891 and 0.3454, which are the slopes representing aflibercept, when placed in F14 from the beginning. From this experiment we can see that the mAb can be recovered by F14, when the mAb has first been treated with PBS.

A pre-exposure test was run in order to test the mAb preservative quality of F14 when challenged with PBS. For this test, ranibizumab, and later aflibercept, was placed into F14 (144 μg/ml). They were then diluted with PBS. A positive control was made using the ranibizumab or aflibercept placed into F14 (144 μg/ml) and then diluted into F14. A negative control was also made by using the ranibizumab or aflibercept placed into PBS (144 μg/ml) and then diluted into PBS.

Table VI shows ranibizumab in PBS, at the higher concentrations, has a lower recovery, with a slope of 0.2026. However, when the ranibizumab/F14 is introduced to the PBS, the recovery is strong with a slope of 0.2505. This value is close to the slope of 0.2158, which is the slope representing ranibizumab, when placed in F14 from the beginning. From this experiment, it can be seen that the mAb can be protected by F14 when the mAb has first been treated with formula 14 and then diluted with PBS.

**Table VI:** Results of Pre-Exposure Study Showing Slopes of Ranibizumab and Aflibercept

Anti-VEGF Agent	PBS Control HPLC Slope	Formula 14 Control HPLC Slope	Formula 14 exposure with PBS diluent HPLC Slope
Ranibizumab			
70-1000 ng/ml	0.1272	0.1971	0.2006
1125-9000 ng/ml	0.2026	0.2158	0.2505
Aflibercept			
70-1000 ng/ml	0.0000	0.1832	0.0471
1125-9000 ng/ml	0.1655	0.2121	0.2262

Table VI also shows the protective effect of F14 is more pronounced at the lower concentrations. Here, the PBS alone has a low recovery with a slope of 0.1272. The F14 protected ranibizumab, when diluted into F14, has a much higher recovery with a slope of 0.1971. And, the F14 protected ranibizumab, when diluted into PBS, has an even higher recovery with a slope of 0.2006. This means that F14 can be used as a primary diluent which protects the mAbs from aggregating. Subsequent dilution with PBS or F14 may then be accomplished with no further loss of mAbs.

The effect of F14 pre-exposure on aflibercept is also shown in Table VI. A pre-exposure test was run in order to test the protective effect of F14. The PBS alone at higher concentrations shows aflibercept with a slope of 0.1655. The F14 and F14 diluted in PBS have very similar slopes of 0.2121 and 0.2262 respectively. Looking at the lower dilutions, the aflibercept in PBS has disappeared, whereas aflibercept pre-exposed with F14 and diluted in PBS has a slope of 0.0471. When aflibercept is placed in F14 and then diluted in F14, the recovery is better with a slope of 0.1832. From this experiment we can see that the fusion protein can be protected by F14, when the fusion protein has first been treated with F14 and then diluted with PBS.

## Discussion

We used SE-HPLC and ELISA as the methods for mAb quantification; SE-HPLC to quantify the monomer amount and ELISA to determine the antibody concentration by assessing the biological functionality of the mAbs and quantifying their biological activity. Additionally, SE-HPLC methods were optimized to quantitate mAb monomer species (the Active Pharmaceutical Ingredient (API)) at very low concentrations, in order to be able to split samples in the range for ELISA validation. In this way, identical samples were analyzed with both methods. This required the SE-HPLC sensitivity and quantification to be within the very low linear range of ELISA. In order to achieve this, we maximized the analytical conditions and parameters for monomers detected by SE-HPLC pursuant to correlation with ELISA. We used a newly marketed SE-HPLC column designed exclusively for mAbs. We set the UV detector wavelength at 212 nm to maximize sensitivity. We then optimized the mobile phase to detect quantities as low as 2.19 ng/ml of monomer. Aggregate peak(s) were lost or greatly diminished with the increased sensitivity to the mAb monomeric content in samples tested (examples of chromatograms are shown in the Supplemental Section).

In order to quantitate any analyte, a reliable analytical standard is needed. We were able to stabilize the monomeric mAb and derive a linear relationship between known concentration dilutions and SE-HPLC peak areas with our system. Column sensitivity between monomers and aggregates further complicates aggregate quantification; while increasing amounts of monomers decreases column sensitivity to aggregates in the same solution (23) The monomeric mAb peak (indicating the amount of active drug) and resulting linear relationship between concentration and peak area, along with the slope of the peak area, serves as the primary indicator of the monomer amount in the solution.

The rationale for this strategy was twofold: (1) HPLC is a fast and low cost method, generally used as the analytical assay to handle large amounts of samples generated by permeation studies, as well as having a wide dynamic range; (2) Elisa kits are highly sensitive, but are generally time consuming, expensive for mAbs, not as accurate as HPLC and have a much narrower dynamic range - necessitating sample dilution to bring into usable range, which can add additional inaccuracy. For these reasons SE-HPLC was anticipated to be our main method of mAb quantification.

We encountered the problem of dilute solution instability while developing and validating methods for the photokinetic enhancement of transscleral mAb permeation, using an *in vitro* Franz diffusion model. Little is known about monoclonal antibody dynamics in dilute solutions. Generally, monoclonal antibodies (mAbs), are formulated in high concentrations (>1 mg/ml, e.g., 1-25 mg/ml, or 10-50 mg/mL), which provide for long term storage stability. This includes bevacizumab, ranibizumab and aflibercept, which are formulated in concentrations of 1 mg/ml or higher, for intraocular injections.

Due to our planned work with Franz diffusion cells and tissue permeation, we needed to develop methods for working with dilute solutions of mAbs. To briefly explain, the Franz cell diffusion technique is an *in vitro* tissue permeation evaluation system frequently used in formulation development. The Franz cell apparatus consists of two primary chambers separated by a membrane. (24) The test compound is applied to the membrane via the top chamber. The bottom chamber contains fluid from which samples are taken at regular intervals for analysis. This testing determines the amount of compound that has permeated the membrane at each time point.

Published tissue permeation (Franz) cell studies typically use PBS as the recipient media at 37°C. Franz cell studies start with a recipient media concentration of 0 of the test compound. As the permeation experiment progresses; the concentration of the test compound increases. However, at early points in the tissue permeation experiment, the drug is still a very dilute solution (i.e., at 2 h, it can be anywhere between 0 and 5000 ng/ml). For our experimental work on anti-VEGF mAbs, we found that these conditions contributed to mAb aggregation, causing reduced monomer concentrations and decreased VEGF binding capacity.

Very dilute solutions of antibodies are also used when creating standard curves and determining human fluid sample quantification by ELISA analytic assay. High sensitivity ELISA methods require drug samples to be diluted to bring the samples within the usable dynamic range of the method, generally 1-1000 ng/ml, more typically 1-100 ng/ml. Frequently, the sample is diluted with PBS, which we found may result in up to 50% loss of the biopharmaceutical agent. Because dilution with PBS decreases monomer concentration, reported drug concentrations may be significantly lower than actual drug concentrations in various *in vitro* and *in vivo* studies.

Antibody aggregation in certain solutions can occur very rapidly. High antibody concentration aggregation (>50 mg/ml) is widely known and is an important issue in antibody manufacturing and packaging for clinical or laboratory use. Low concentration antibody aggregation is generally controlled in the immunoassay environment through the use of added serum proteins (human or bovine serum albumen i.e. BSA). Clinically, serum derived protein additives, animal or human, are generally disfavored or unacceptable for administration into humans. Therefore, the ability to prevent aggregation in certain immunoassays and drug delivery formulations by using diluent reagents that are free of extraneous serum/proteins, which may cause non-specific antigen binding or provide cross reactivity of certain antibodies to multiple antigens, would be of great benefit.

We have identified a phenomenon wherein low concentrations of antibodies (<1150 ng/ml), in PBS or the manufacturer’s formula, lose monomer concentration. This phenomenon has recently been reported by others as well. (25) The discovery of mAb monomer reduction is particularly important in clinical settings, wherein dilute antibody formulations are administered. One such example would be in oncology IV treatment, wherein the mAb formulation is diluted in a saline IV bag. Dilution also occurs in AMD treatment, when an anti-VEGF antibody is administered at 50 μl of a 10-40 mg/ml solution (0.5 mg) into the viscous vitreous humor of the eye – which is generally around 4 ml.

The human vitreous humor contains about 95% water, insoluble collagens, glycosaminoglycans (hyaluronic acid, chondroitin sulfate and heparin sulfate), metabolites, amino acids, fatty acids, prostaglandins, cells and enzymes. The total soluble protein concentration in the vitreous body is between 0.02% and 0.14% (200-1400 μg/ml), 40% of which is albumin (80-560 μg/ml, 0.008%–0.056%). (26) The relatively low concentration of total soluble proteins and albumin in the vitreous body may offer little protection from antibody aggregation within the vitreous body compared to the normal total soluble protein in the circulating blood which is 6.4–8.3% with 3.5–5% of that portion being albumin.

Typically, antibody based immunoassays use about 1% serum albumin soluble protein to retard antibody aggregation at their low concentrations. Clinically, any added albumin into an antibody composition would be pharmacologically unacceptable for human administration due to possible immune interactions.

In the case of an intravitreal injection, the vitreous of the eye has a concentration gradient from 10 to 40 mg/ml at the injection deposition site toward a 0 mg/ml concentration throughout the adjacent vitreous. A low concentration zone precedes the drug front as it slowly permeates throughout the vitreous volume. The low concentration drug front has conditions that promote low concentration antibody aggregation. Once antibodies aggregate, dis-aggregation is difficult or impossible without some form of intervention.

As stated above, our strategy was to use SE-HPLC as a high throughput method for the determination of mAb concentrations in our permeation experiments. We first needed to correlate and validate SE-HPLC with ELISA in order to show equivalency.

ELISA provides a method that indicates functionality of a subject antibody which is used to determine drug concentration. The antibody or subject drug must be functional to initiate binding in the assay. SE-HPLC provides a quantification of a particular molecular size of a compound but does not indicate functionality; only the quantity of a particular molecular weight entity in a solution. Samples of the same subject drug/ carrier matrix were evaluated by splitting the same sample and simultaneously analyzing the samples by ELISA and SE-HPLC and comparing the derived concentration for the concentration gradient slope.

Validation and agreement were performed by Pearson product-moment correlation, wherein a linear correlation between matched data sets derived from the two analytical methods can be evaluated. The closer the derived Pearson correlation factor is to 1.0 the closer the correlation. Statistical significance wherein *p* < 0.05 between the data set values demonstrates that the Pearson correlation cannot be a random association and that the correlation is statistically significant. In our studies, shown in Fig. 3b–d, close and significant correlation between ELISA and SE-HPLC analytical methods validate SE-HPLC as a method to evaluate different formulation compositions over the wide dynamic range of the SE-HPLC method. Based on these findings, SE-HPLC was used as the analytical technique for a majority of the subsequent studies.

The derived slope value of a known concentration serial dilution sequence provides a mathematical function that can be interpolated to determine an unknown sample concentration. This is the basis of most analytical chemistry quantification. Derived ELISA optical density and derived SE-HPLC area under the curve are examples of values that can be used to make a standard dilution curve and provide a mathematical function to quantify the concentration in an unknown sample. The slope value is an indication of the sensitivity of the method. For example, a slope value of 0.5 demonstrates a higher sensitivity than a slope of value 0.005. If all analytical test conditions remain constant for a given subject compound except for a difference in the carrier matrix that the compound is formulated, then a comparison of the slopes of two different carrier matrix formulation can be evaluated to determine if there is a difference in the formulation matrix. (27) Based on the plotted serial dilution curves, drug carrier matrix effects can be expressed as a comparison of the dilution curve slopes. If the slope of one carrier matrix is less than a comparator, then the carrier matrix with the lower slope value expresses carrier matrix suppression. In other words, the higher the slope value, the higher the protective properties of the carrier matrix. Therefore, SE-HPLC can be used to evaluate antibody drug matrix effects to determine carrier matrix formulations that have better anti-aggregation potential.

**Fig. 4:**
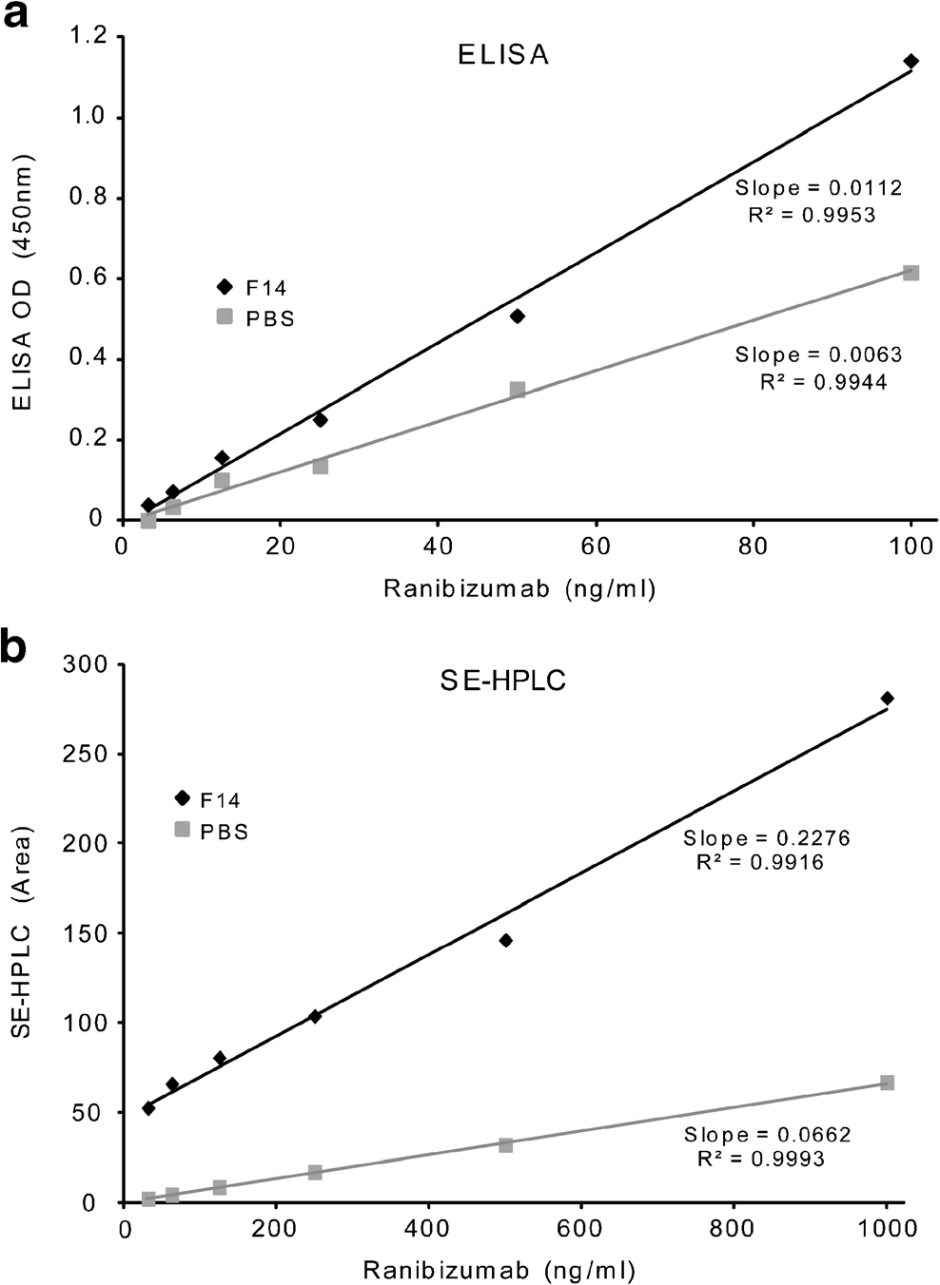
Comparison of the two analytical methods ELISA (**a**) and SE-HPLC (**b**) for ranibizumab diluted with Formula 14 and PBS.

Single area peak values derived from SE-HPLC, report essentially the monomeric antibody concentration, as the higher order of aggregates formed would pass through the size exclusion column and elute at different time points and not be enumerated. In contrast in immunoassays, aggregated antibodies may still have a substrate binding potential depending on the exact location of the antibody to antibody aggregation point. So long as an antigen binding site is unobscured, antigen binding may occur in aggregated situations however at a reduced amount relative to the total number of antibodies in the carrier matrix. ELISA methods measure the number of antigen/antibody binding occurrences. As SE-HPLC single peak area determinations excludes aggregated antibody forms, ELISA optical density determination will include some binding from otherwise aggregated antibodies. In this sense, the comparative slope value ratio of two analytical methods for the same solution may give different slope ratios for SE-HPLC *versus* ELISA as both methods measure monomeric antibody concentrations while ELISA may additionally report the influence of aggregated antibodies. For example, using ranibizumab as a model drug, Fig. 4a shows the ratio of ELISA slopes of formula 14 *versus* PBS diluent slope of 1.77 (0.0112/0.0063) whereas Fig. 4b shows the SE-HPLC slope ratio is 3.43 (0.2276/0.0662). The difference in the slope ratios of the two testing methods for the same antibody carrier matrices may be due to the SE-HPLC method measuring only monomer species while the ELISA method may include antigen/antibody with monomers in addition to other aggregated antibody forms.

Frequently, biological fluid samples, containing antibodies, are diluted with phosphate buffered saline (PBS) to bring the antibody concentration into the linear range of an ELISA method. In this case, PBS dilution may result in up to 50% loss of the biopharmaceutical agent. Because dilution with PBS increases aggregation, reported drug concentrations in biological samples may be significantly lower than actual drug concentrations in various *in vitro* and *in vivo* immunoassay studies.

Bevacizumab, ranibizumab and aflibercept, as the primary anti-VEGF agents utilized by transscleral delivery, are very prone to aggregation and diminished function once removed from the manufacturer’s vial and diluted. For example, as shown in Fig. 1a, we found that monomeric bevacizumab significantly decreased when phosphate buffered saline (PBS) was used as the diluent. Additionally, it was found that the manufacturer’s formulations for ranibizumab, in regards to the storage and delivery of the agents, did not protect the drugs from aggregation under dilution and elevated temperatures that would be encountered in *in vitro* and *in vivo* experimentation (Fig. 1b). We also noticed, surprisingly, that there was a concentration discontinuity between 1000 ng/ml and 1150 ng/ml (see Fig. 3a). This concentration discontinuity was evident and consistent throughout all of our studies with the three anti-VEGF agents.

A stable formulation, one that preserves the monomeric mAb, is required to keep the molecular mass and molecular radius small enough to allow for topical delivery through the sclera and maintain biological activity in the vitreous. Formulation and delivery issues for monoclonal antibody therapeutics have been reviewed. (5) Our aim was to identify a formulation that protected the mAb monomer. This protective quality, for dilute concentrations, would be reflected in a higher SE-HPLC slope value, as well as the reduction of the discontinuity between 1000 ng/ml and 1150 ng/ml. Based upon the manufacturer’s formulations, excipient substitutions were screened with dilutions of standard concentrations of ranibizumab to test various carrier matrices. Formulations, shown in Table I, were tested using SE-HPLC.

Water, PBS, saline (0.9% NaCl) and 0.3% NaCl were used as the starting solutions. Other than the manufacturer’s formulation, a sodium phosphate buffer system was used in all of the formulations for pH control. Trehalose was used throughout the formulations, owing to its protein stabilizing quality. The amino acids arginine, histidine and glutamic acid were tested in various capacities.

Histidine is commonly used as a stabilizer in mAb formulations. We incorporated histidine into our early formulations; however we did not see any improvement in the SE-HPLC slope or reduction of the slope discontinuity. We did start to see improvement when we switched to 10 mM arginine.

It has been suggested that arginine, particularly L-arginine, may be toxic to the eye tissue when used in an intravitreal injection ophthalmic formulation. (28,29) In these studies, the assumed damage was dose dependent. Lower doses showed no complications. The concentration of arginine used was 34 mg/ml or 200 mM. Subsequent investigations of other compounds with arginine (30,31), which cite these two articles, also showed that retinal damage was dose dependent. Lower doses showed no damage. The concentration of arginine used in Loewenstein *et al.* was 50 mg/ml. Rowely *et al.* used 52.2 mg/ml of arginine in their stock solution. Another study of tissue plasminogen activator (32), suggests that the L-arginine in the vehicle increases nitric oxide and intracellular cyclic–GMP. This is compelling but not conclusive. The study was run on a cell culture basis, which is an artificial system without vascular flow, as opposed to an *in vivo* study which is high perfusion and has high circulatory turnover. The concentration of arginine used was 35 mg/ml or 200 mM.

These studies seem to suggest that there is toxicity due to arginine when dosed at high concentrations. Low doses of tPA, which include arginine, appear to be safe. This fact is further supported by another study which looked at retinal tolerance to bevacizumab in co-application with a recombinant tissue plasminogen activator. Lüke *et al.* found that in the control arm of their study that “no evidence for toxic effects on the function of photoreceptors or the higher neuronal network was detected when testing the concentration of the solvent carrier, which was equivalent to a concentration of 20mg/ml r-tPA.” (33).

The intended use for our formulation is for a topically applied ocular dose which is then held for 1 h and irradiated with concurrent light for transscleral delivery. The arginine concentration in our formulation is 1.74 mg/ml or 10 mM. This is a much lower concentration than the 34 mg/ml or 200 mM amount used in the cited studies above. Additionally, the length of time for transscleral dosage application is 1 h, once or twice per week, not continuously for a month - as would be found in an intravitreal injection. We expect any small amount of arginine that would permeate the sclera would then be further diluted in the vitreous, making the retinal tissue exposure to arginine extremely small. Therefore, with the concentration of arginine being very small in the topically applied dose, and which is applied for a short time, we think that arginine in F14 is acceptable for ophthalmic applications.

Surfactants were also tested. The manufacturer’s formulation of ranibizumab contained 0.01% Tween 20. We increased the percent from 0.01% to 0.04% and then switched from Tween 20 to Tween 80 (low peroxide). (34) For comparison, Avastin has 0.04% Tween 20, Lucentis has 0.01% Tween 20 and IAI Eylea has 0.03% Tween 20. Our Tween 80 (low peroxide) surfactant concentration of 0.04% is in the range of the other 3 commercially available anti-VEGF agent formulations. Therefore, since our surfactant level is similar to, and no more, than marketed products, we hypothesize that our improved results are due to a synergistic effect of the excipients rather than simply a higher surfactant level protecting against adsorption.

Formula 14 was then tested for its compatibility in ELISA. Drug standard dilutions, using F14, had higher slope values (2X) when compared to slope values generated from the ELISA diluting reagent for bevacizumab, ranibizumab and aflibercept. The ELISA diluting reagent for all 3 mAbs contains PBS and BSA. The higher slopes indicated that F14 did not interfere with ELISA assays (data not reported).

Additionally, there appears to be no pH dependence of when using F14. SE-HPLC slopes for the 3 compositions (Formula 14 at pH 6.78, 7.0, and 7.4) were essentially the same, as shown in Table III. Aflibercept was tested with the same 3 compositions. Again, no difference was observed between F14 at the 3 pH values. This demonstrates that F14 overcomes narrow ranges of usable pH. Furthermore F14 can be used as a diluent for mAb compositions at a physiologic pH of 7.4.

Several of the formulations, with ranibizumab, were then tested for stability. As seen in Table IV, the manufacturer’s formulation, F1, shows a discontinuity between 1000 ng/ml and 1150 ng/ml. When using F14, the critical concentration point at 1000 to 1150 ng/ml is much less evident before and after exposure to 37°C. Note the significantly higher slope values when compared to the manufacturer’s formulation. When ranibizumab is diluted to low concentrations <1125 ng/ml in manufacturer’s formulation, there is an immediate 40% loss of drug activity, when compared to F14. Heating to body temperature significantly produces more degradation in other formulations, but not F14.

In Franz cell permeation studies, described earlier, the receiver solution is degassed to prevent bubbles from forming in the receiver chamber. If the solution were not degassed, the 37°C heating and the stirring by magnetic stir bar would release dissolved air causing bubbles to form under the study tissue surface, thereby impeding drug transport through the tissue and into the receiver media. We investigated the effects of degassing on ranibizumab solutions (see Supplemental Section), and confirmed that partial degassing formulation solutions significantly improved the stability of these agents (Fig. S1). When F14 was degassed and tested against non-degassed F14 and PBS, the degassed formula was 5% higher in recovery than non-degassed formula and 44% higher in recovery than the PBS solution.

It is our hypothesis that formulating bevacizumab, ranibizumab and aflibercept requires the step of degassing the protein solution. The mechanism of aggregation may be due to the formation of dissolved gas bubble bridges between two or more antibodies. Hydrophobic areas on the antibody surface are considered to be the most probable point of antibody aggregation. Dissolved air can manifest as nanobubbles (35,36), having diameters 40 – 300 nm, which can act as a bubble bridge between the hydrophobic areas of proteins, thereby creating aggregates. It is believed that degassing partially removes dissolved air that would otherwise adhere to hydrophobic areas. The synergistic effect of the degassed solution with the formulation of surfactant, carbohydrate and amino acid allows the mAb to move freely within the solution without aggregating due to bubble to bubble contact or surface adsorbed nanobubbles from the solution. F14 excipients may coat the hydrophobic surface areas of the antibodies and eliminate or reduce the attachment of nanobubbles to those hydrophobic areas, thereby reducing aggregation potential.

An antibody stabilization carrier matrix formulation may have usefulness in antibody production and pre-packaging steps prior to final packaging for distribution. It would be important and advantageous to have a carrier matrix that exhibited the potential to reverse or dis-aggregate antibody aggregates in a solution. A dis-aggregation medium would be useful in upstream antibody manufacturing (cell culture harvest) as well as downstream processing (i.e. filtration, concentration and packaging) to increase production yield and reduce process losses.

## Conclusion

The goal of our work was to develop methods in order to successfully conduct in-vitro photokinetic transscleral mAb delivery and to demonstrate the permeation enhancement of mAbs due to light irradiation. Pursuant to this objective, we discovered the intrinsic potential for antibody aggregation of anti-VEGF therapeutics; which renders topical therapy all but impossible. We found that when Avastin^®^, Lucentis^®^ and IAI Eylea^®^ are diluted in PBS or with the manufacturer’s formulation there is a 40–50% loss of monomer concentration and loss of drug binding activity at low concentrations. Whether this loss is due to antibody-surface adsorption, antibody-antibody coupling, a combination of both, or some other mechanism, we needed to develop a formulation that would protect the mAb monomer at very low and low concentrations, for 24 h and at 37°C.

Monoclonal antibody drugs tend to aggregate causing the activity to be greatly diminished. Various excipients are added to stabilize the drug for extended shelf life. Dilution and brief exposure to body temperatures quickly cause aggregation and diminished drug activity. When bevacizumab and ranibizumab are diluted in the same formulation used by the manufacturer, there is an immediate 40% loss of drug activity. Samples diluted with PBS diminish >50% of their activity immediately. ELISA methods use PBS as a diluent to bring samples into method range. Prior research by others, determining drug concentration in human tissue samples as well as *in vitro* permeation studies, may have under-reported drug concentrations.

We have developed a formulation (F14) that significantly protects the mAbs from heat and dilution dependent aggregation. Regardless of the reason for mAb monomer loss, the anti-aggregation formula appears to allow a greater monomer recovery from diluted samples. F14 contains sodium phosphate buffer, 0.3% NaCl, 10% trehalose, 10 mM arginine and 0.04% polysorbate 80 at a pH of 6.78. This formula is also stable at a pH of 7.4. Degassing the formulation provides additional stability and mAb monomer recovery. The formula also allows for the disaggregation of previously aggregated mAb in dilute solutions.

F14 may be useful, for example, when antibody formulations are diluted into normal (0.9%) saline for intravenous administration. F14 would also be useful as administered into the eye vitreous where there is little endogenous protein buffer that may prevent aggregation. The formulation may be of use to mAb manufacturers in the realm of manufacturing and packaging of the drug. The formulation may also be useful in immunoassay development and use in commercial ELISA kit manufacture for multiple mAbs. Finally, the formulation is necessary to properly study drug permeation *in vitro* and may be part of a topical formulation for *in vivo* drug delivery.

### ACKNOWLEDGMENTS AND DISCLOSURES

The authors wish to acknowledge and thank Genentech, South San Francisco CA, for the gift of Lucentis and Regeneron, Tarrytown, NY, for the gift of IAI Eylea. The authors also want to thank Dr. Nathaniel G. Butlin, of United Immunoassay, Inc. for helpful discussions and review of the manuscript. Additional thanks are extended to Drs. Jaafar El Annan, MD and Larry Puthenparambil, MD, University of Texas Medical Branch, Department of Ophthalmology and Visual Sciences, for reviewing this manuscript.

## Electronic supplementary material

ESM 1 (DOC 35 kb)

Fig. S1 Results of a degassing experiment with ranibizumab in Formula 14. (GIF 25 kb)

High Resolution Image (TIFF 3188 kb)

Fig. S2a Bevacizumab 144,000 ng/ml. (GIF 12 kb)

High Resolution Image (TIFF 96 kb)

Fig. S2b Bevacizumab 562.50 ng/ml. (GIF 42 kb)

High Resolution Image (TIFF 366 kb)

Fig. S2c Standard curve of bevacizumab: 4.3945 ng/ml – 144,000 ng/ml. (GIF 33 kb)

High Resolution Image (TIFF 9049 kb)

Fig. S3a Ranibizumab 144,000 ng/ml. (GIF 15 kb)

High Resolution Image (TIFF 85 kb)

Fig. S3b Ranibizumab 562.50 ng/ml. (GIF 51 kb)

High Resolution Image (TIFF 393 kb)

Fig. S3c Standard curve of ranibizumab: 4.3945 ng/ml – 144,000 ng/ml. (GIF 31 kb)

High Resolution Image (TIFF 8520 kb)

Fig. S4a Aflibercept 144,000 ng/ml. (GIF 15 kb)

High Resolution Image (TIFF 147 kb)

Fig. S4b Aflibercept 562.50 ng/ml. (GIF 28 kb)

High Resolution Image (TIFF 389 kb)

Fig. S4c Standard curve of aflibercept: 4.3945 ng/ml – 144,000 ng/ml. (GIF 30 kb)

High Resolution Image (TIFF 8256 kb)

Table S1 (GIF 47 kb)

High Resolution Image (TIFF 14 kb)

Table S2 (GIF 119 kb)

High Resolution Image (TIFF 34 kb)

Table S3 (GIF 37 kb)

High Resolution Image (TIFF 3660 kb)

Table S4 (GIF 38 kb)

High Resolution Image (TIFF 3661 kb)

Table S5 (GIF 32 kb)

High Resolution Image (TIFF 3180 kb)

## Abbreviations

AMD: Age related macular degeneration
BSA: Bovine serum albumen
ELISA: Enzyme-linked immunosorbent assay
IV: Intravenous
mAb: Monoclonal antibody
PBS: Phosphate buffered saline
SE-HPLC: Size exclusion high-performance liquid chromatography
tPA: Tissue plasminogen activator
VEGF: Vascular endothelial growth factor
