# Supplementary material for: Formulation Stabilization and Disaggregation of Bevacizumab, Ranibizumab and Aflibercept in Dilute Solutions
Source: Pharm Res. 2018 Feb 28;35(4):78. doi: 10.1007/s11095-018-2368-7 (PMC5830485; doi:10.1007/s11095-018-2368-7)
Supplement: Supplementary file 1 — (DOC 35 kb) [file 11095_2018_2368_MOESM1_ESM.doc]

Supplemental Material

Formulation Stabilization and Disaggregation of Bevacizumab, Ranibizumab and Aflibercept in Dilute Solutions

*Steven A. Giannos*, Edward R. Kraft, Zhen-Yang Zhao, Kevin H. Merkley, Jiyang Cai*

*University of Texas Medical Branch, Department of Ophthalmology and Visual Sciences, Galveston, Texas*

**Fraction/Percent of Manufacturer's Composition after Dilution with PBS**

Supplementary Table I

Table S 1

**Formulation Preparation**

Based upon the manufacturer's formulation, excipient substitutions were screened with dilutions of standard concentrations of ranibizumab. Formula 1 was the manufacturer’s formulation prepared in a 1L quantity. Formula 2 – 14 were 1L preparations, with excipients added or deleted, as shown in Table 1.

Briefly, in a 1 liter volumetric flask, while stirring at room temperature, trehalose, phosphates, sodium chloride and other components are added to about 850ml of DI water and stirred. After all solids are dissolved, the total volume is brought up to 999.6 ml with water. pH is adjusted by adding additional phosphate as need to achieve the desired pH. After all solids are dissolved, 400μl of Polysorbate (Tween® 20 or Tween® 80) is added. The solution is stirred to ensure adequate mixing. This solution is then filtered through 0.22μm filter system and degassed.

**Standard Serial Dilutions**

In some experiments, serial dilutions for ELISA and SE-HPLC analysis were made as follows: A starting concentration of the subject antibody, as provided by the manufacturer (bevacizumab 25mg/ml and ranibizumab 10mg/ml), was diluted volume to weight (v/w) with the subject diluent matrix to a concentration of 100µg/ml (100,000ng/ml). This 100µg/ml was then diluted volume to volume (v/v) to 10000ng/ml as a starting concentration. The 10000ng/ml solution was diluted v/v to 6000ng/ml. The 6000ng/ml solution was then diluted volume to volume (v/v) with the subject diluent in steps providing ng/ml concentrations at 3000, 1500, 1250, and 1150. An intermediate dilution of 1000ng/ml was made and that 1000ng/ml solution was serially diluted in 1:1 steps providing 500, 250, 125, 62.5, 31.25, 15.625, 7.8125, 3.90125ng/ml. The 250ng/ml dilution was used for the high standard concentration for bevacizumab ELISA. The 125ng/ml was used for the high standard concentration for the ranibizumab ELISA

In some other experiments, serial dilutions for ELISA and SE-HPLC analysis were made as follows: a starting concentration of the subject antibody as provided by the manufacturer (bevacizumab 25mg/ml, ranibizumab 10mg/ml and aflibercept 48.2mg/ml) was diluted volume to weight (v/w) with the subject diluent matrix to a concentration of 144µg/ml (144,000ng/ml). This starting concentration was then diluted volume to volume (v/v) with the subject diluent in 1: 1 steps providing ng/ml concentrations at 72000, 36000, 18000, 9000, 4500, 2250, 1125, 562.50, 281.25, 140.62, 70.31, 35.156, 17.578, 8.79, 4.39, 2.197 and 1.0985. An intermediate dilution of 1000ng/ml was made from the 2250ng/ml step. To provide certain calibration standard ranges for ELISA method ranges, for example, an additional dilution of 225ng/ml was made from the 2250ng/ml bevacizumab dilution for that ELISA method. An intermediate dilution of 100ng/ml was made from the 1000ng/ml dilution for ranibizumab and for aflibercept for those ELISA methods.

**Fraction of Manufacturer's Formulation After Dilution with F14**

Supplementary Table II

Table S 2

**Degassing Study**

A series of ranibizumab standards was prepared in order to test the issue of degassing the formulation. Briefly, 40ml of Formula 14 were introduced into two, 50ml conical tubes respectively. One tube was then degassed under vacuum, with agitation and stirring, for 1 hour. The other tube was not degassed. Serial standard solutions of ranibizumab, ranging from 3.9ng/ml to 10,000ng/ml, were then prepared from the degassed and non-degassed formula 14. These sets were then analyzed by SE-HPLC.

The results of the degassing experiment are shown in Fig. S1. The standards, when in non-degassed PBS, were low, having a slope of 0.2007. When the standards were prepared with non-degassed formula 14, the recovery increased and the slope was 0.2988. The recovery increased even more when the ranibizumab standards were prepared in degassed formula 14. Here, the slope improves from 0.2988 to 0.3103. Therefore, when Formula 14 was degassed and tested against non-degassed Formula 14 and PBS, the degassed formula was 5% higher in recovery than non-degassed formula and 44% higher in recovery than the PBS solution.

Insert Fig. S1

**SE-HPLC Chromatogram Samples**

**Bevacizumab**

Fig. S 2a

Fig. S 2b

Supplementary Table III

Table S 3

Fig S 2c

**Ranibizumab**

Fig. S 3a

Fig. S 3b

Supplementary Table IV

Table S 4

Fig S 3c

**Aflibercept**

Fig. S 4a

Fig. S 4b

Supplementary Table V

Table S 5

Fig S 4c
