# Supplementary figures and images for: Formulation Stabilization and Disaggregation of Bevacizumab, Ranibizumab and Aflibercept in Dilute Solutions
Source: Pharm Res. 2018 Feb 28;35(4):78. doi: 10.1007/s11095-018-2368-7 (PMC5830485; doi:10.1007/s11095-018-2368-7)

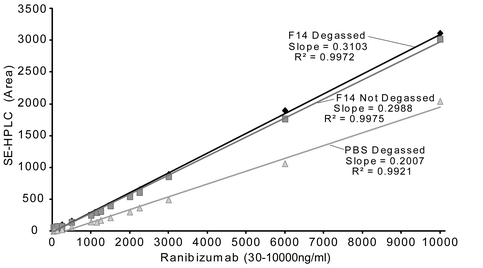

Supplement: Supplementary file 2 — Results of a degassing experiment with ranibizumab in Formula 14. (GIF 25 kb) [file 11095_2018_2368_Fig5_ESM.gif]

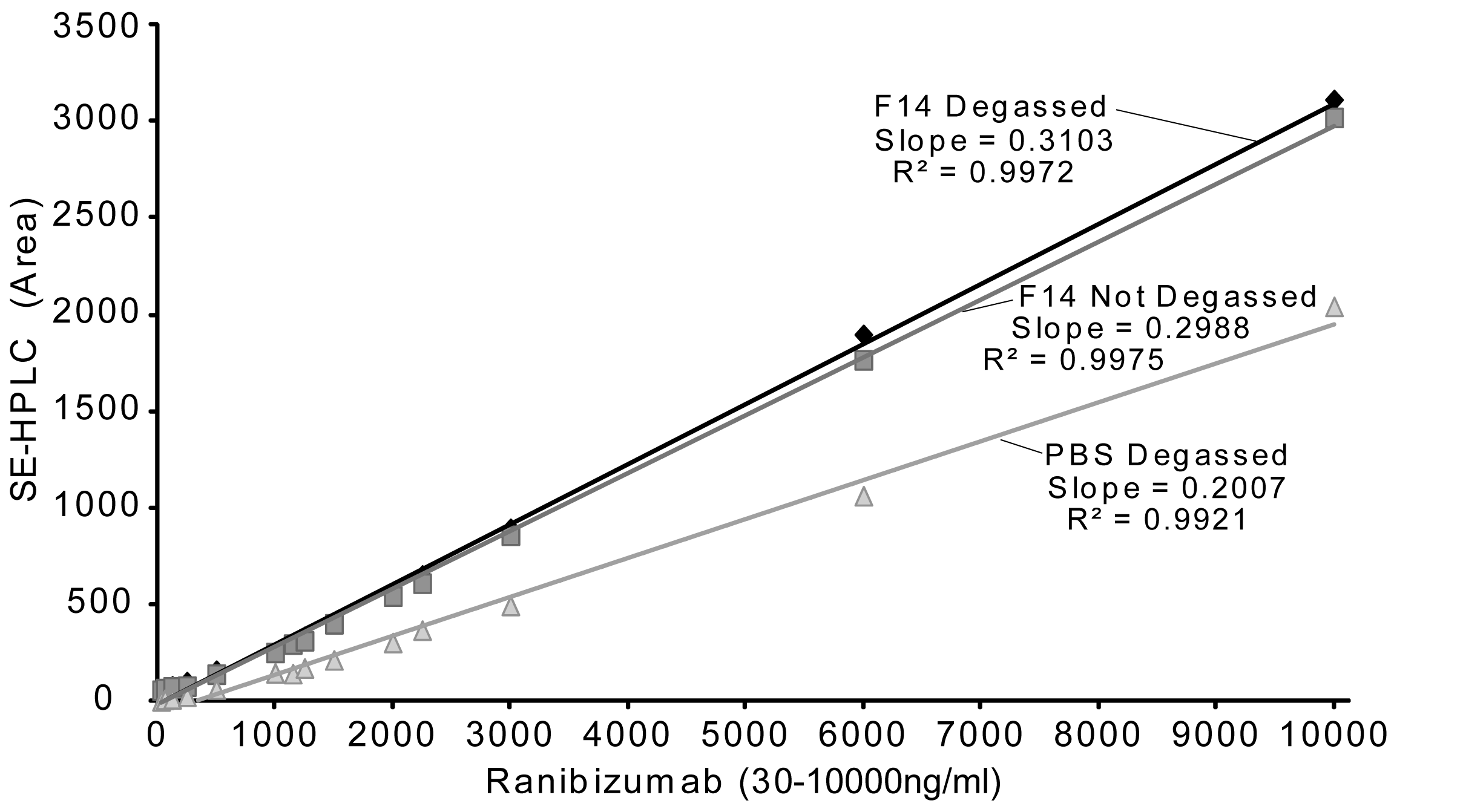

Supplement: Supplementary file 3 — High Resolution Image (TIFF 3188 kb) [file 11095_2018_2368_MOESM2_ESM.tif]

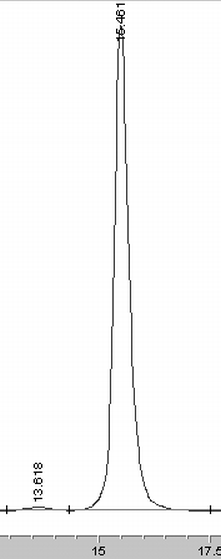

Supplement: Supplementary file 4 — Bevacizumab 144,000 ng/ml. (GIF 12 kb) [file 11095_2018_2368_Fig6_ESM.gif]

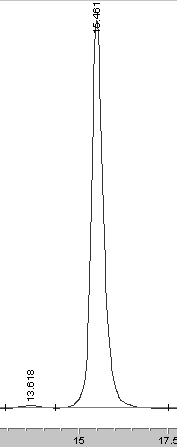

Supplement: Supplementary file 5 — High Resolution Image (TIFF 96 kb) [file 11095_2018_2368_MOESM3_ESM.tif]

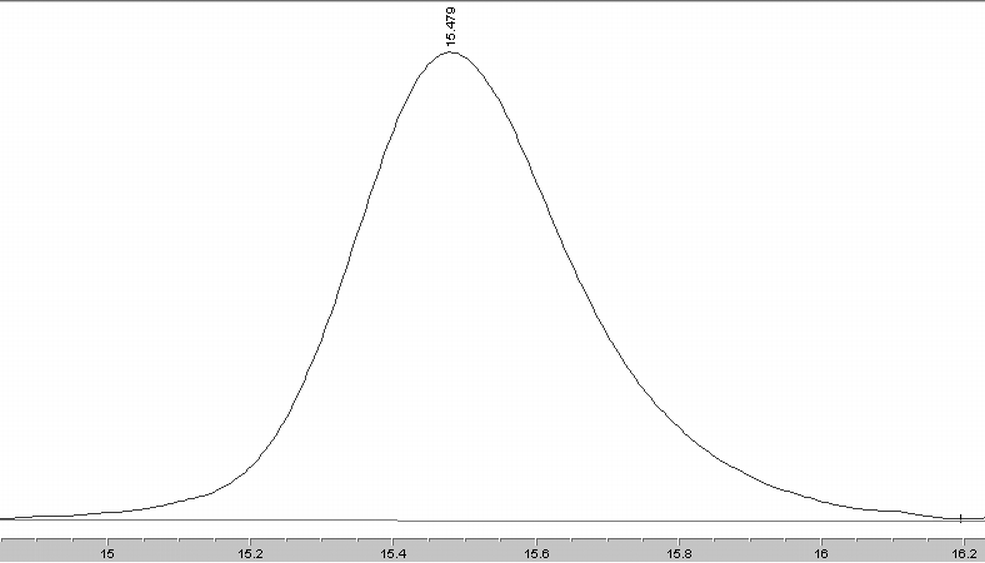

Supplement: Supplementary file 6 — Bevacizumab 562.50 ng/ml. (GIF 42 kb) [file 11095_2018_2368_Fig7_ESM.gif]

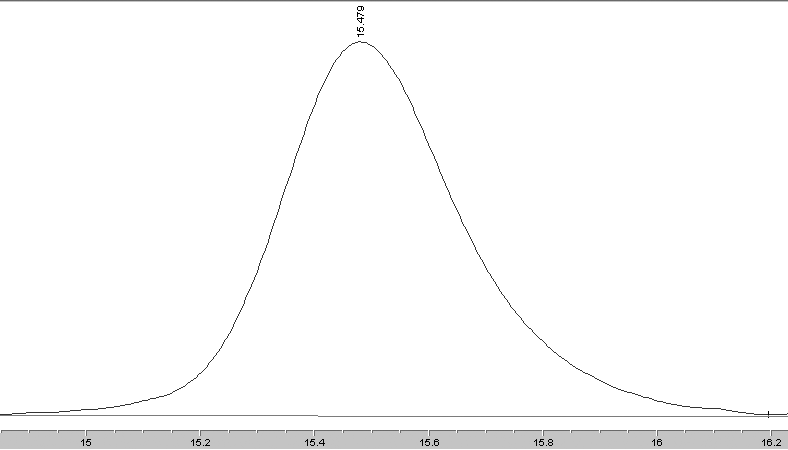

Supplement: Supplementary file 7 — High Resolution Image (TIFF 366 kb) [file 11095_2018_2368_MOESM4_ESM.tif]

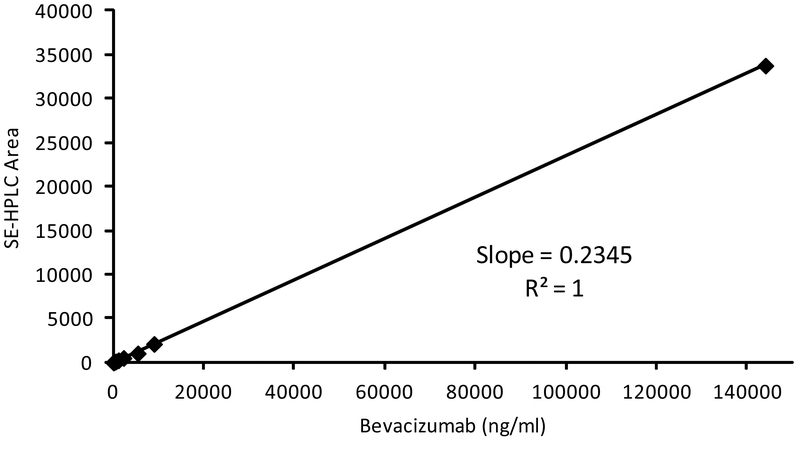

Supplement: Supplementary file 8 — Standard curve of bevacizumab: 4.3945 ng/ml – 144,000 ng/ml. (GIF 33 kb) [file 11095_2018_2368_Fig8_ESM.gif]

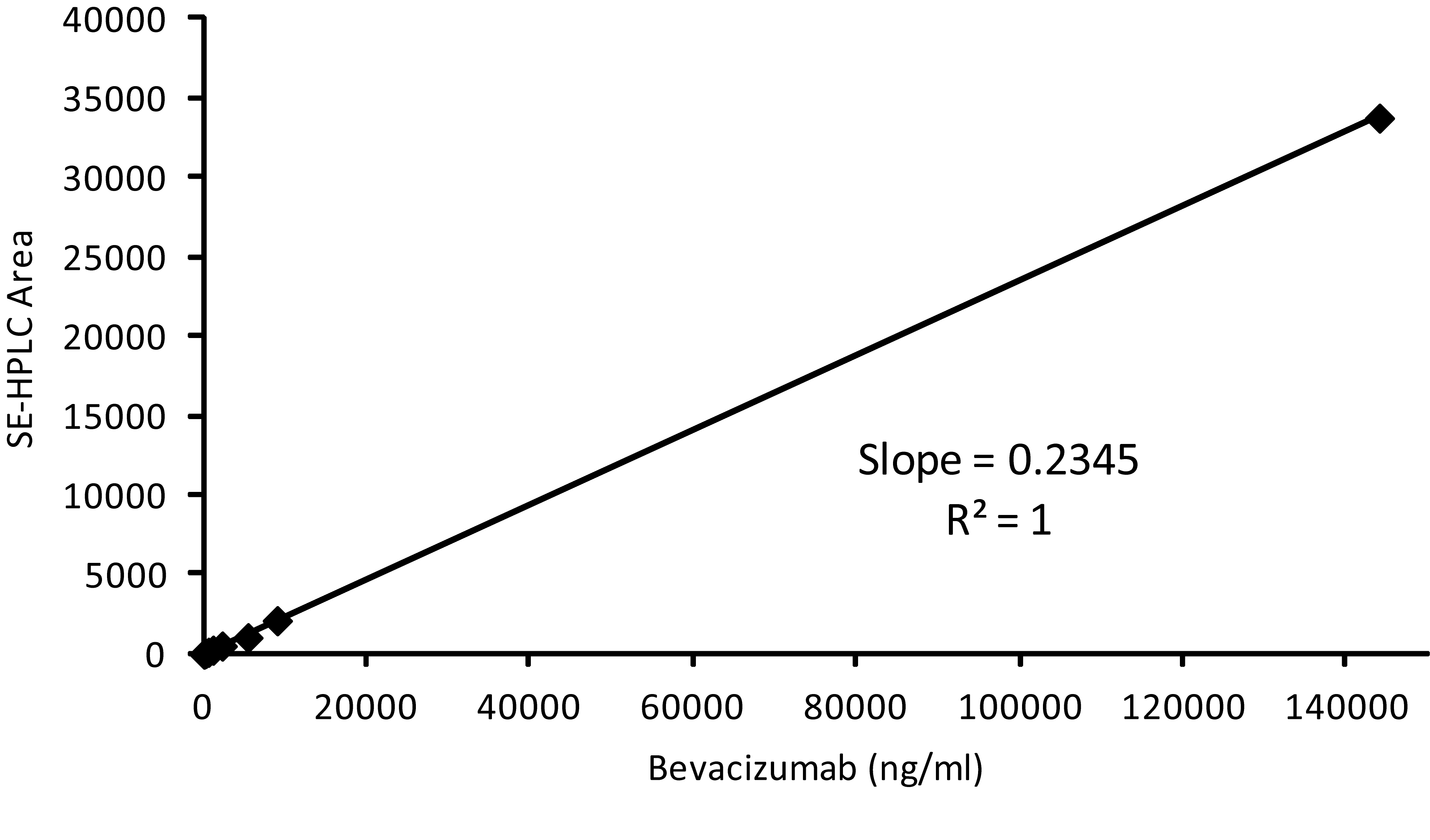

Supplement: Supplementary file 9 — High Resolution Image (TIFF 9049 kb) [file 11095_2018_2368_MOESM5_ESM.tif]

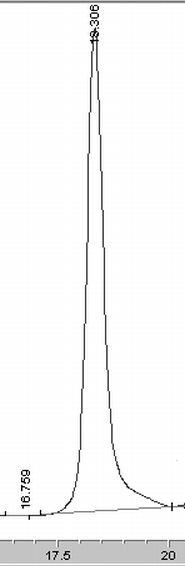

Supplement: Supplementary file 10 — Ranibizumab 144,000 ng/ml. (GIF 15 kb) [file 11095_2018_2368_Fig9_ESM.gif]

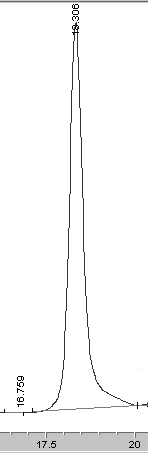

Supplement: Supplementary file 11 — High Resolution Image (TIFF 85 kb) [file 11095_2018_2368_MOESM6_ESM.tif]

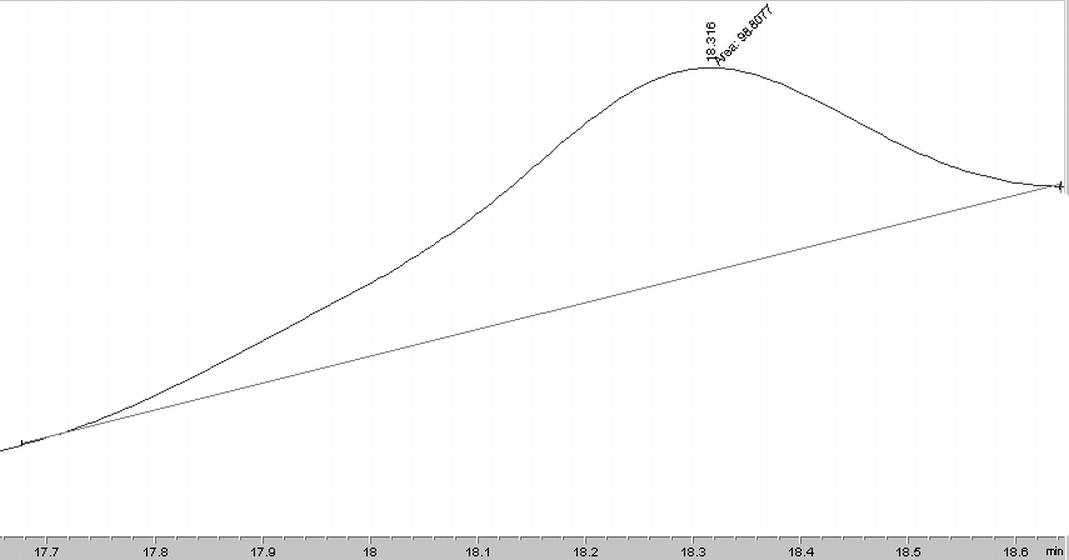

Supplement: Supplementary file 12 — Ranibizumab 562.50 ng/ml. (GIF 51 kb) [file 11095_2018_2368_Fig10_ESM.gif]

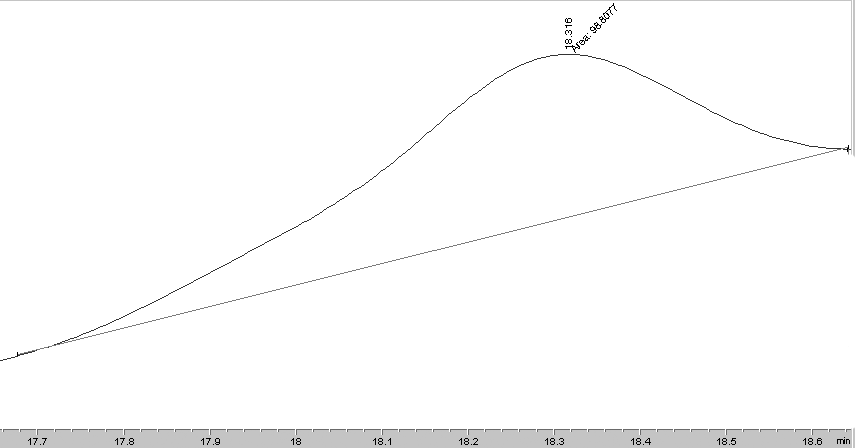

Supplement: Supplementary file 13 — High Resolution Image (TIFF 393 kb) [file 11095_2018_2368_MOESM7_ESM.tif]

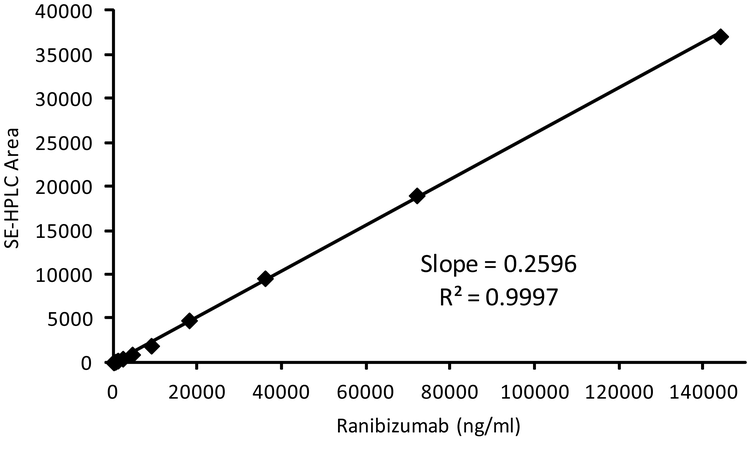

Supplement: Supplementary file 14 — Standard curve of ranibizumab: 4.3945 ng/ml – 144,000 ng/ml. (GIF 31 kb) [file 11095_2018_2368_Fig11_ESM.gif]

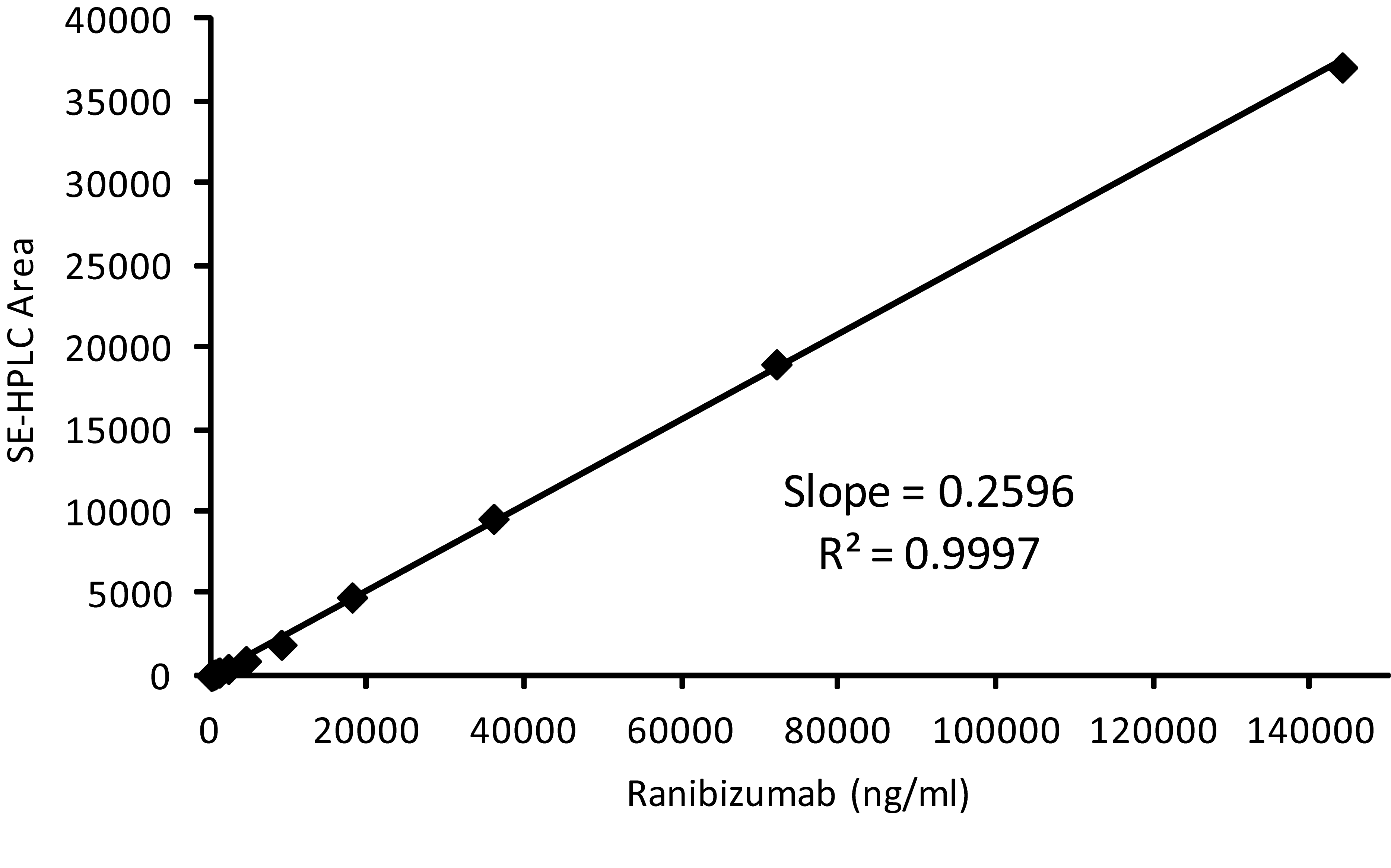

Supplement: Supplementary file 15 — High Resolution Image (TIFF 8520 kb) [file 11095_2018_2368_MOESM8_ESM.tif]

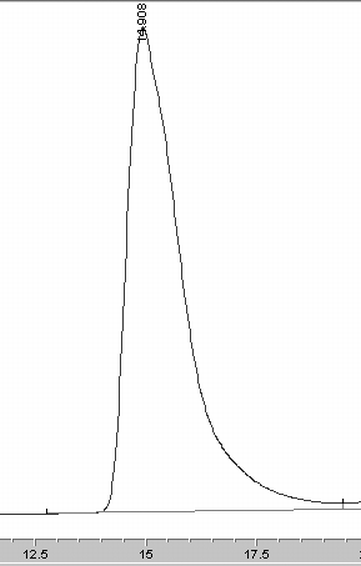

Supplement: Supplementary file 16 — Aflibercept 144,000 ng/ml. (GIF 15 kb) [file 11095_2018_2368_Fig12_ESM.gif]

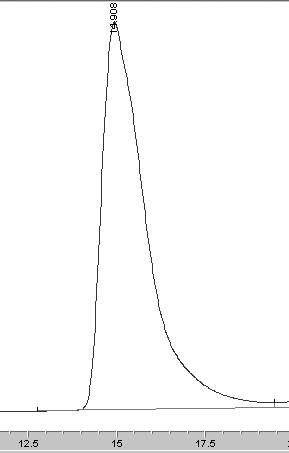

Supplement: Supplementary file 17 — High Resolution Image (TIFF 147 kb) [file 11095_2018_2368_MOESM9_ESM.tif]

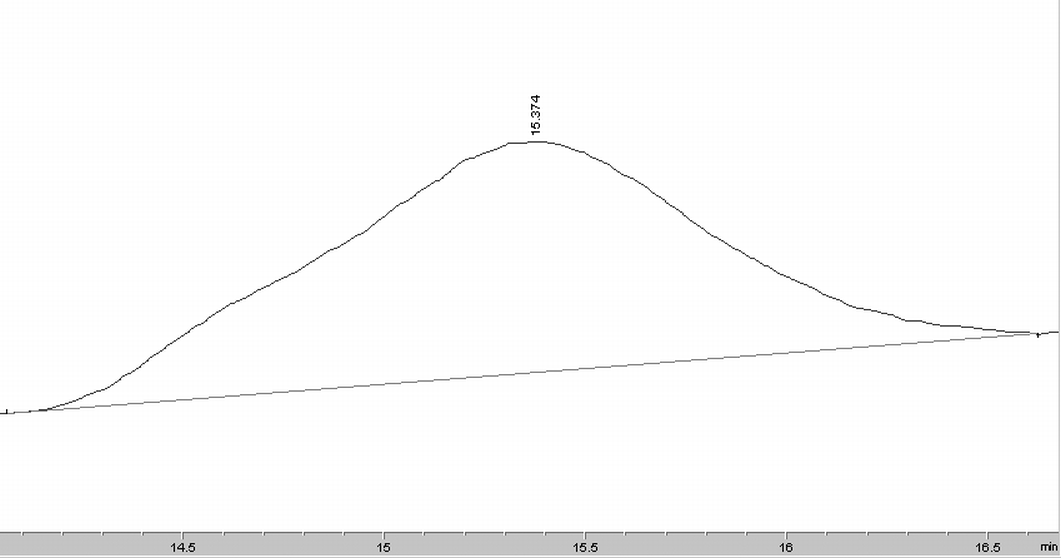

Supplement: Supplementary file 18 — Aflibercept 562.50 ng/ml. (GIF 28 kb) [file 11095_2018_2368_Fig13_ESM.gif]

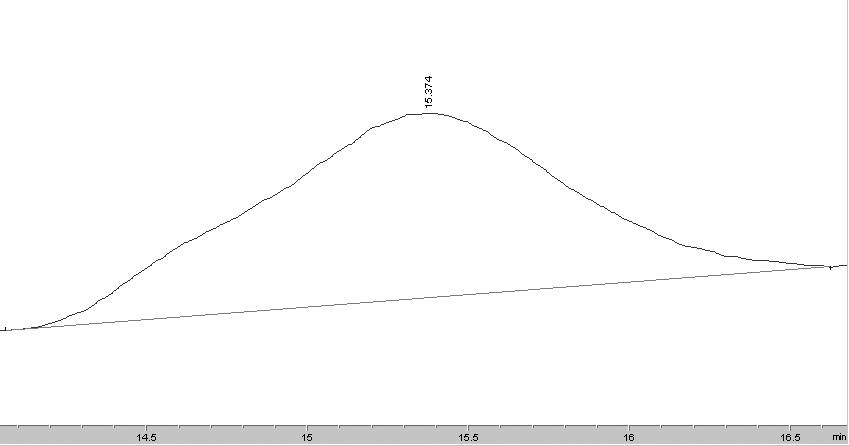

Supplement: Supplementary file 19 — High Resolution Image (TIFF 389 kb) [file 11095_2018_2368_MOESM10_ESM.tif]

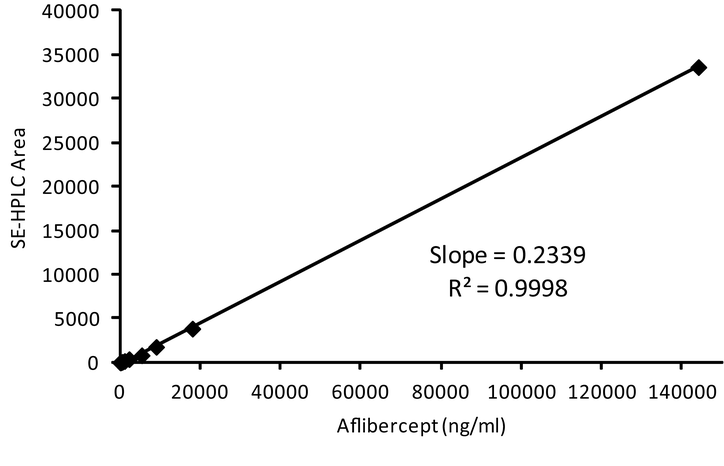

Supplement: Supplementary file 20 — Standard curve of aflibercept: 4.3945 ng/ml – 144,000 ng/ml. (GIF 30 kb) [file 11095_2018_2368_Fig14_ESM.gif]

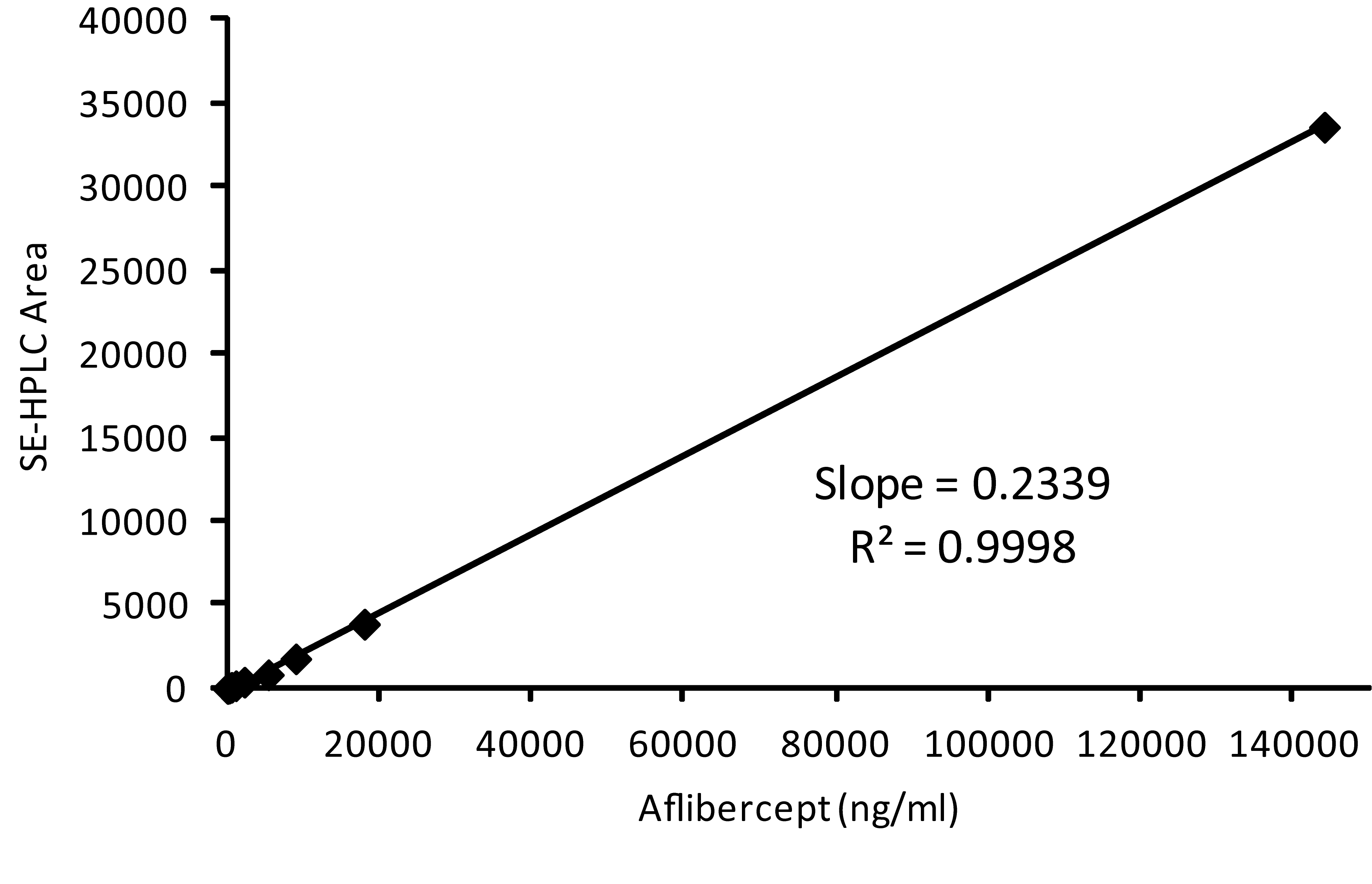

Supplement: Supplementary file 21 — High Resolution Image (TIFF 8256 kb) [file 11095_2018_2368_MOESM11_ESM.tif]

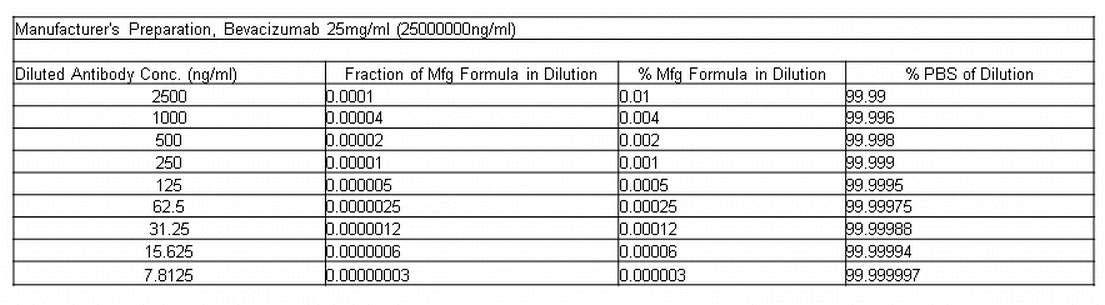

Supplement: Supplementary file 22 — (GIF 47 kb) [file 11095_2018_2368_Fig15_ESM.gif]

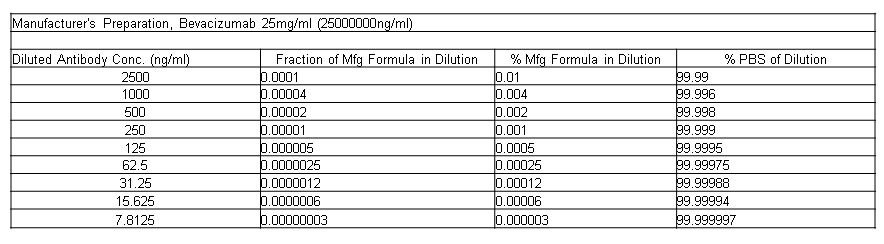

Supplement: Supplementary file 23 — High Resolution Image (TIFF 14 kb) [file 11095_2018_2368_MOESM12_ESM.tif]

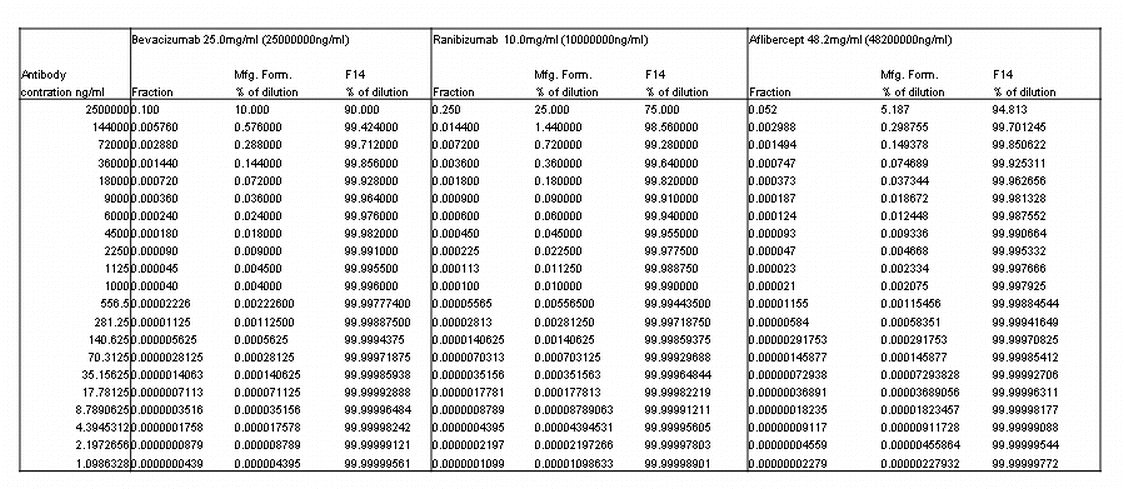

Supplement: Supplementary file 24 — (GIF 119 kb) [file 11095_2018_2368_Fig16_ESM.gif]

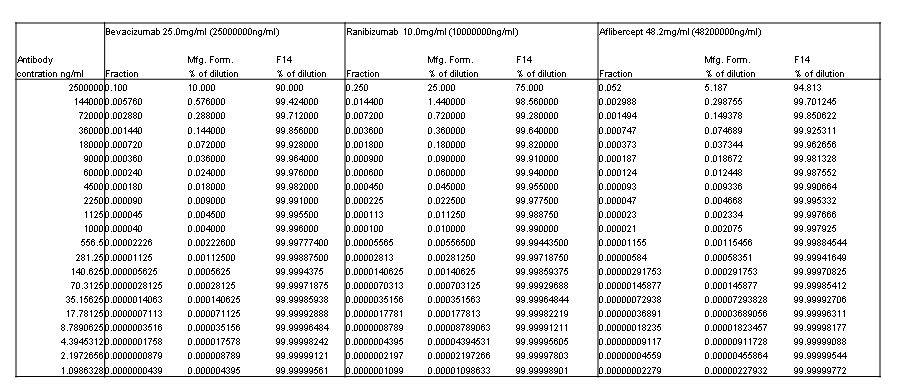

Supplement: Supplementary file 25 — High Resolution Image (TIFF 34 kb) [file 11095_2018_2368_MOESM13_ESM.tif]

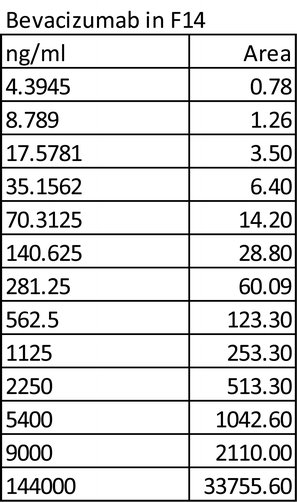

Supplement: Supplementary file 26 — (GIF 37 kb) [file 11095_2018_2368_Fig17_ESM.gif]

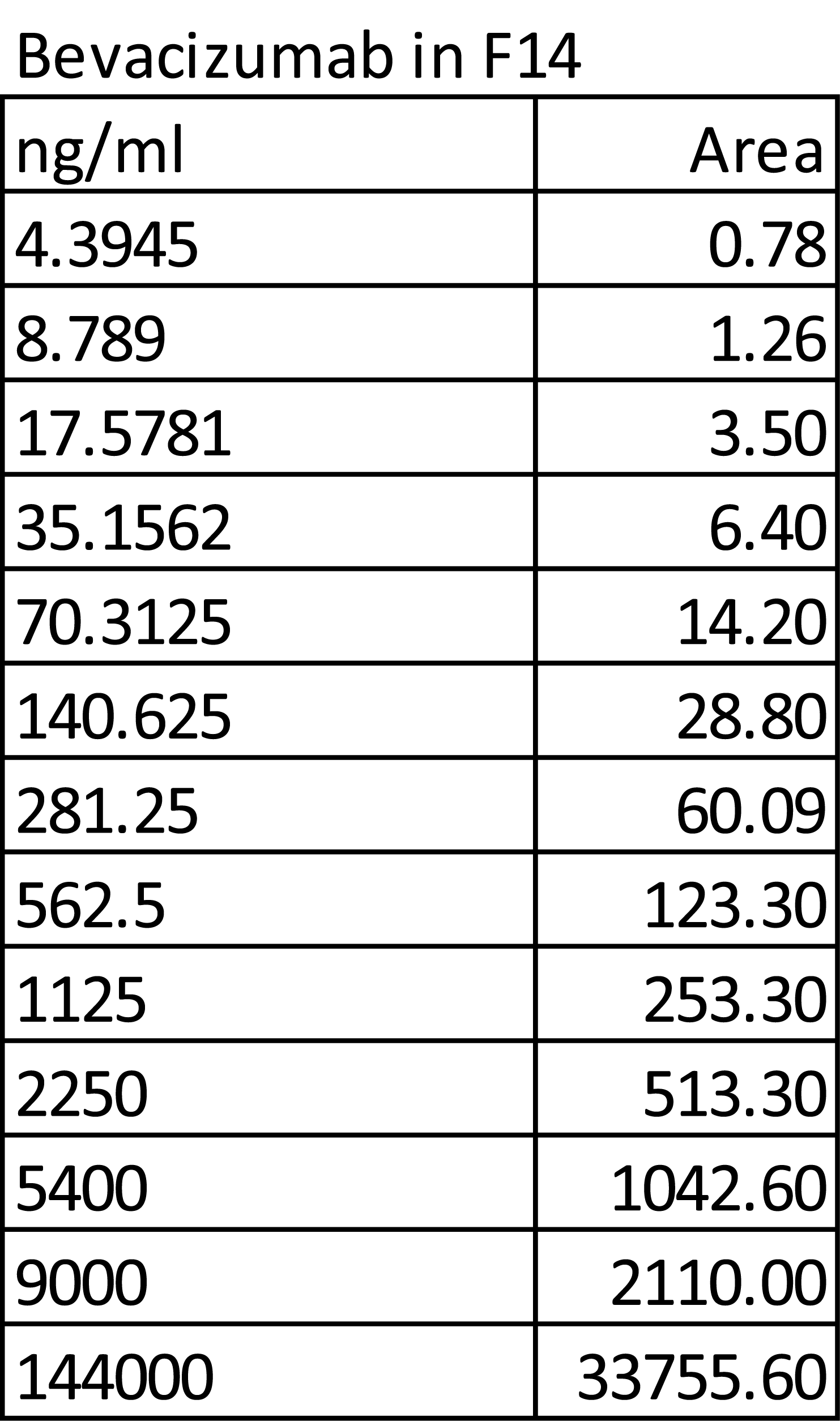

Supplement: Supplementary file 27 — High Resolution Image (TIFF 3660 kb) [file 11095_2018_2368_MOESM14_ESM.tif]

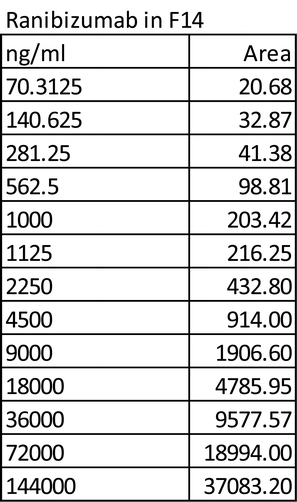

Supplement: Supplementary file 28 — (GIF 38 kb) [file 11095_2018_2368_Fig18_ESM.gif]

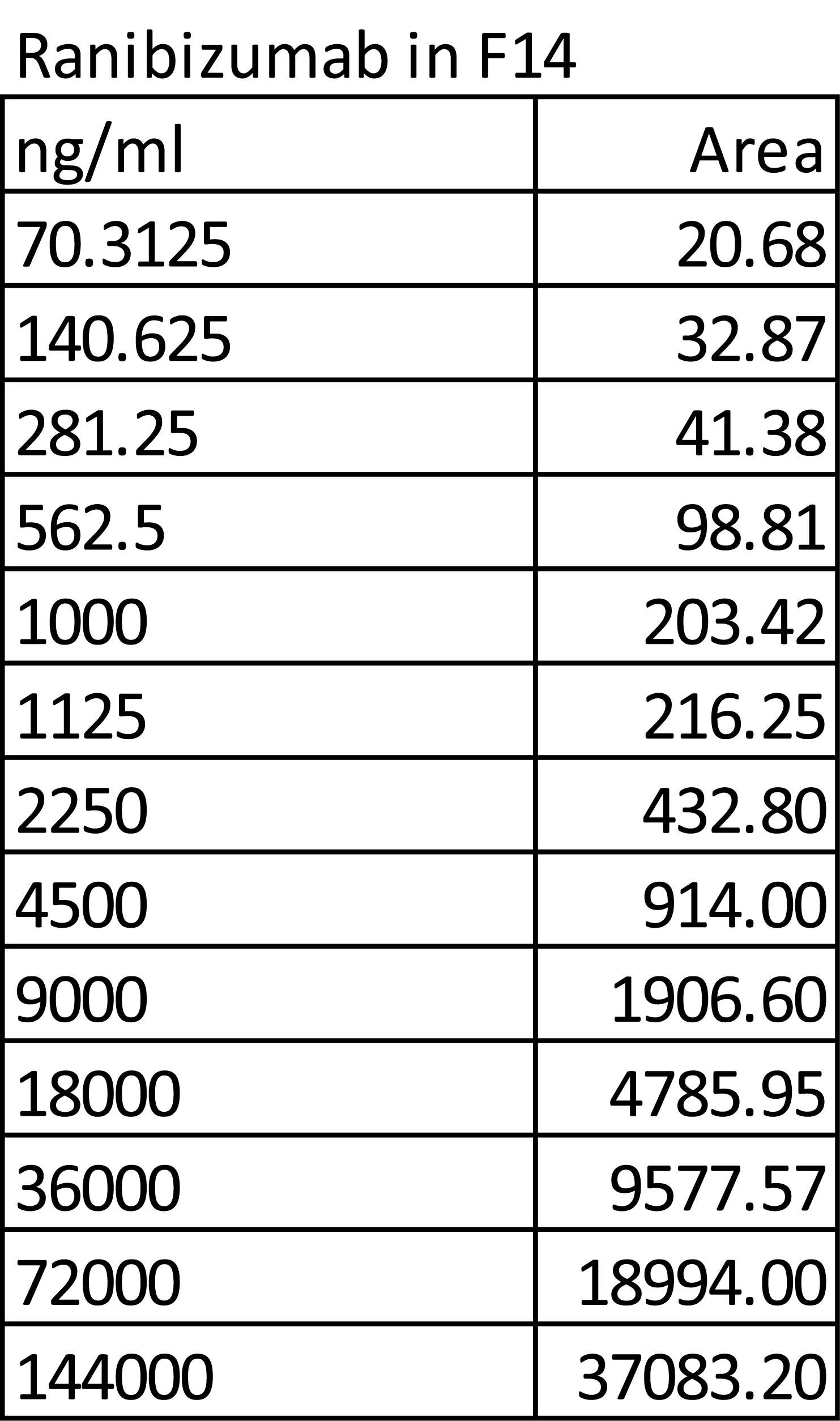

Supplement: Supplementary file 29 — High Resolution Image (TIFF 3661 kb) [file 11095_2018_2368_MOESM15_ESM.tif]

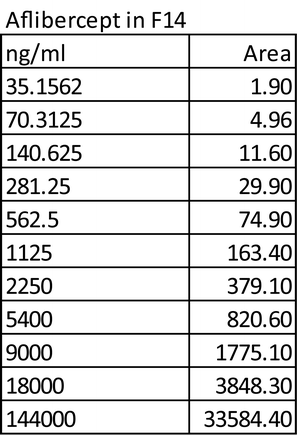

Supplement: Supplementary file 30 — (GIF 32 kb) [file 11095_2018_2368_Fig19_ESM.gif]

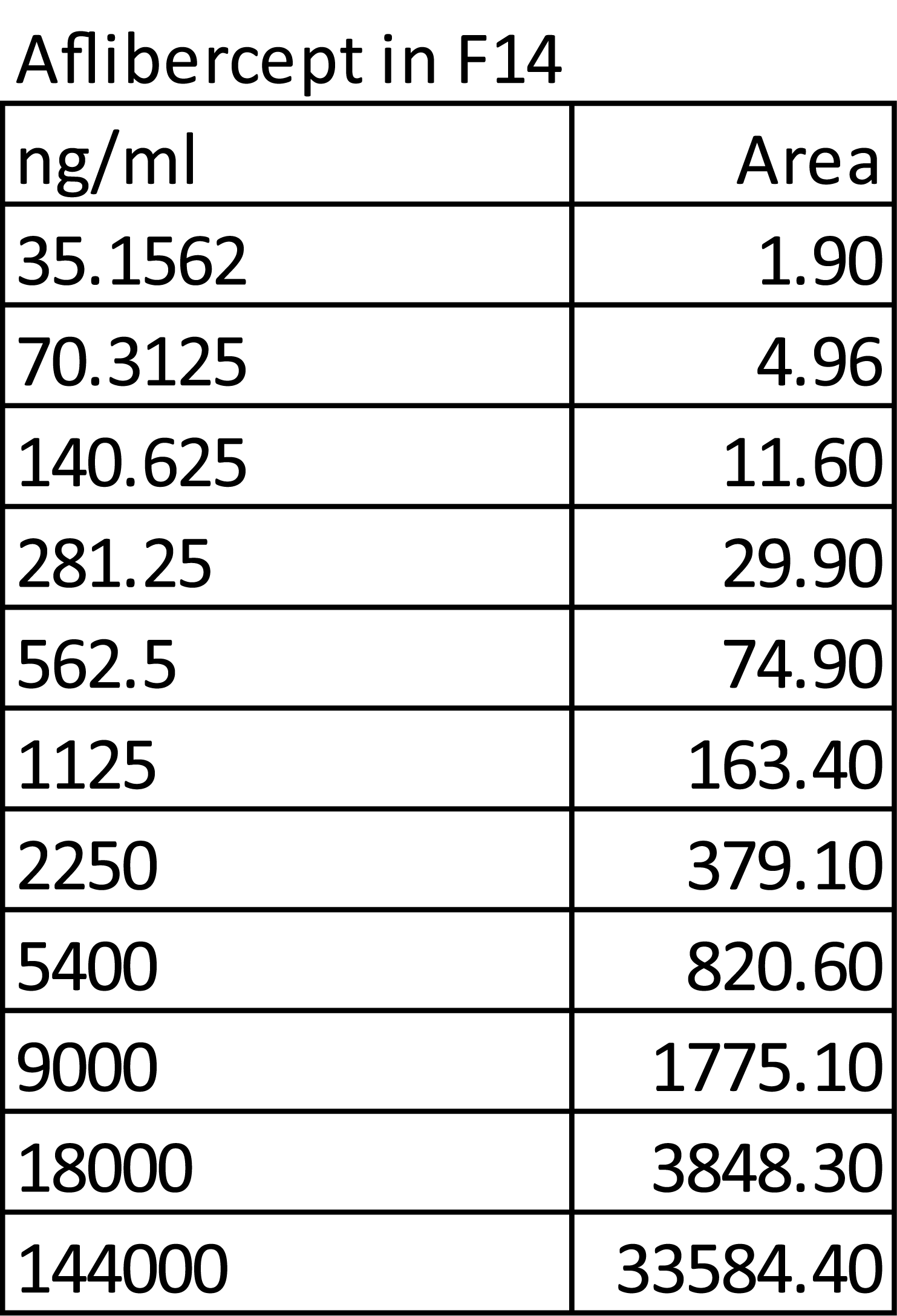

Supplement: Supplementary file 31 — High Resolution Image (TIFF 3180 kb) [file 11095_2018_2368_MOESM16_ESM.tif]
